# Supplementary material for: De novo assembly of a young Drosophila Y chromosome using single-molecule sequencing and chromatin conformation capture
Source: PLoS Biol. 2018 Jul 30;16(7):e2006348. doi: 10.1371/journal.pbio.2006348 (PMC6117089; doi:10.1371/journal.pbio.2006348)
Supplement: S1 Fig — The chromosomal scaffolds (after Hi-C scaffolding) and unscaffolded contigs are demarcated by dotted lines and ordered based on their female-to-male coverage ratio. Each dot represents the average coverage across a 50-kb window. (PDF) [file pbio.2006348.s001.pdf]

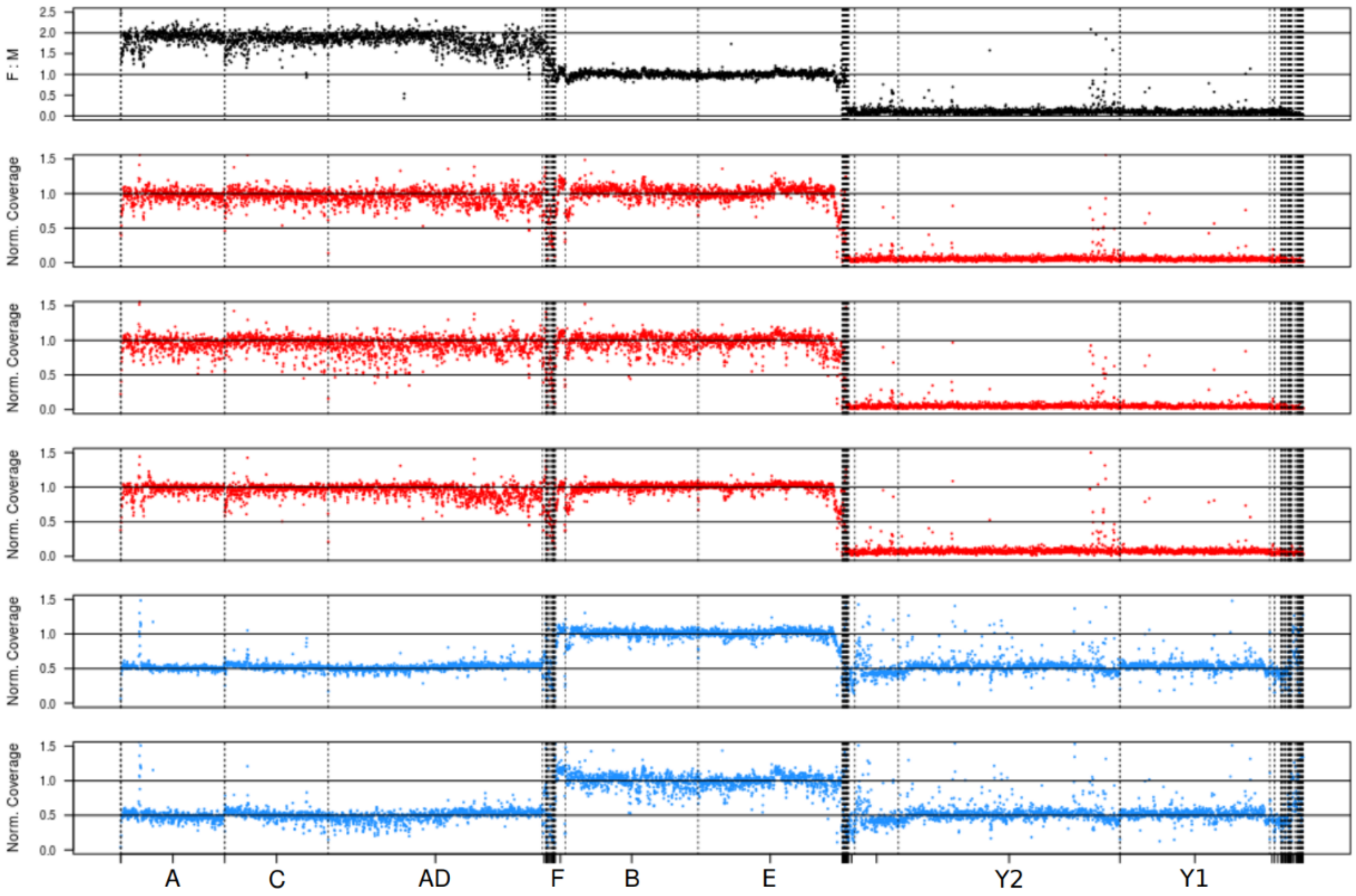

S1 Fig – Illumina sequencing coverage of three individual females (red) and males (blue), and the female-to-male coverage ratio (black). The chromosomal scaffolds (after Hi-C scaffolding) and un-scaffolded contigs are demarcated by dotted lines and ordered based on their female-to-male coverage ratio. Each dot represents the average coverage across a 50-kb window.
