# Supplementary material for: De novo assembly of a young Drosophila Y chromosome using single-molecule sequencing and chromatin conformation capture
Source: PLoS Biol. 2018 Jul 30;16(7):e2006348. doi: 10.1371/journal.pbio.2006348 (PMC6117089; doi:10.1371/journal.pbio.2006348)
Supplement: S2 Fig — The Hi-C association heatmap of the PacBio contigs (demarcated by dotted lines), sorted by contig size (left panels), is reorganized using 3D-DNA (middle panels), generating near-chromosome-length scaffolds (black boxes, right panels). (PDF) [file pbio.2006348.s002.pdf]

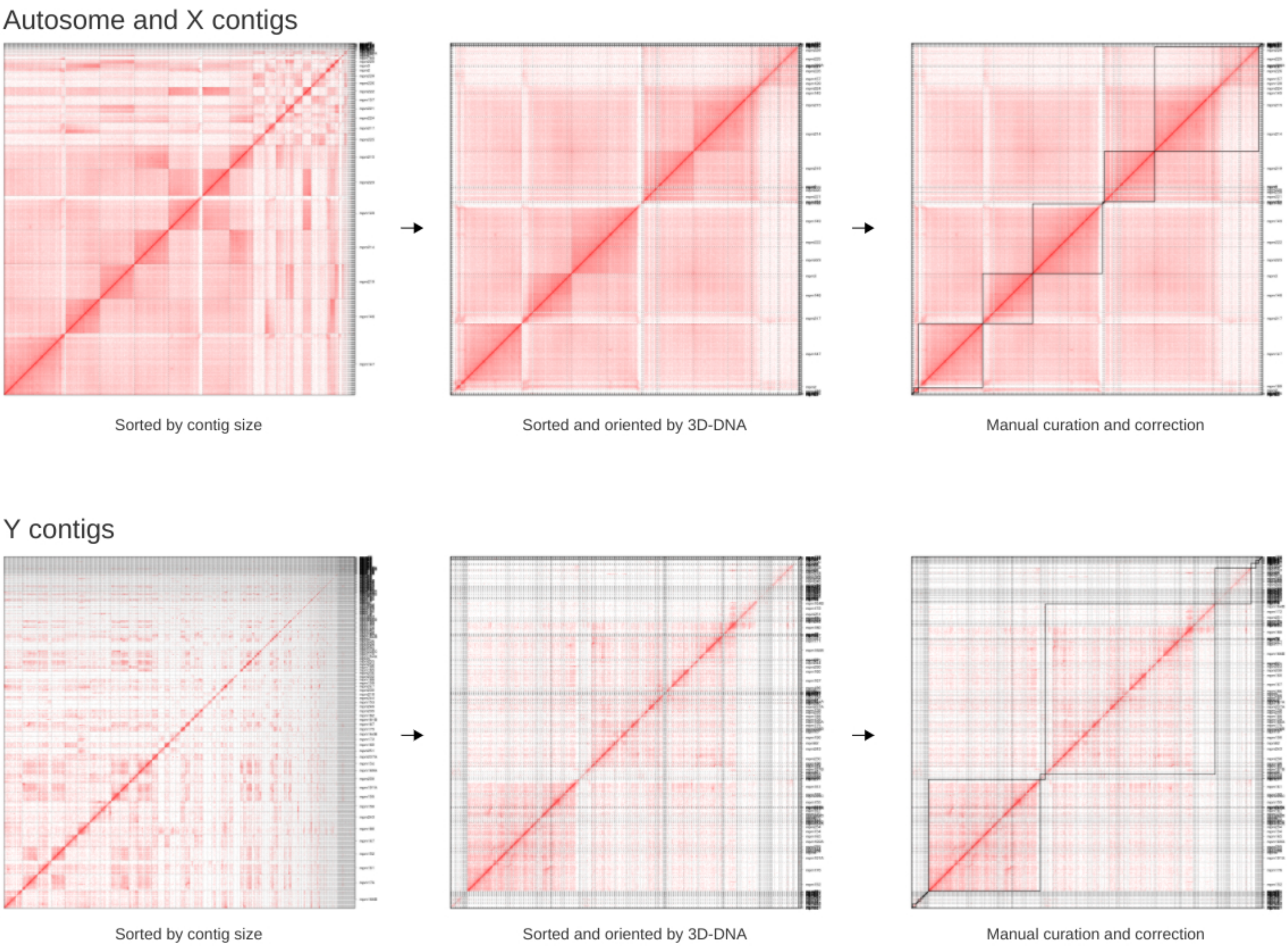

S2 Fig – Hi-C association density maps for autosomes and X contigs, and Y contigs.
The Hi-C association heatmap of the Pacbio contigs (demarcated by dotted lines), sorted by contig size (left panels) is reorganized using 3D-DNA (middle panels), generating near chromosome length scaffolds (black boxes, right panels).
