# Supplementary material for: De novo assembly of a young Drosophila Y chromosome using single-molecule sequencing and chromatin conformation capture
Source: PLoS Biol. 2018 Jul 30;16(7):e2006348. doi: 10.1371/journal.pbio.2006348 (PMC6117089; doi:10.1371/journal.pbio.2006348)
Supplement: S3 Fig — Hi-C linkage density map, gene and repeat content for A. chromosome XL, B. chromosome XR, C. chromosome 2, D. chromosome 4, E. neo-X chromosome, F. chromosome YD, G. neo-Y_1, H. neo-Y_2, I. Muller F. Neo-Y_1 and neo-Y_2 refer to the two largest neo-Y scaffolds (see Fig 2C). Note that regions of increased repeat density (such as centromeres or the repeat islands on chromosome 2 and 4) show increased contact probabilities with other repeats. Chromosome arms/scaffolds are not drawn to scale. (PDF) [file pbio.2006348.s003.pdf]

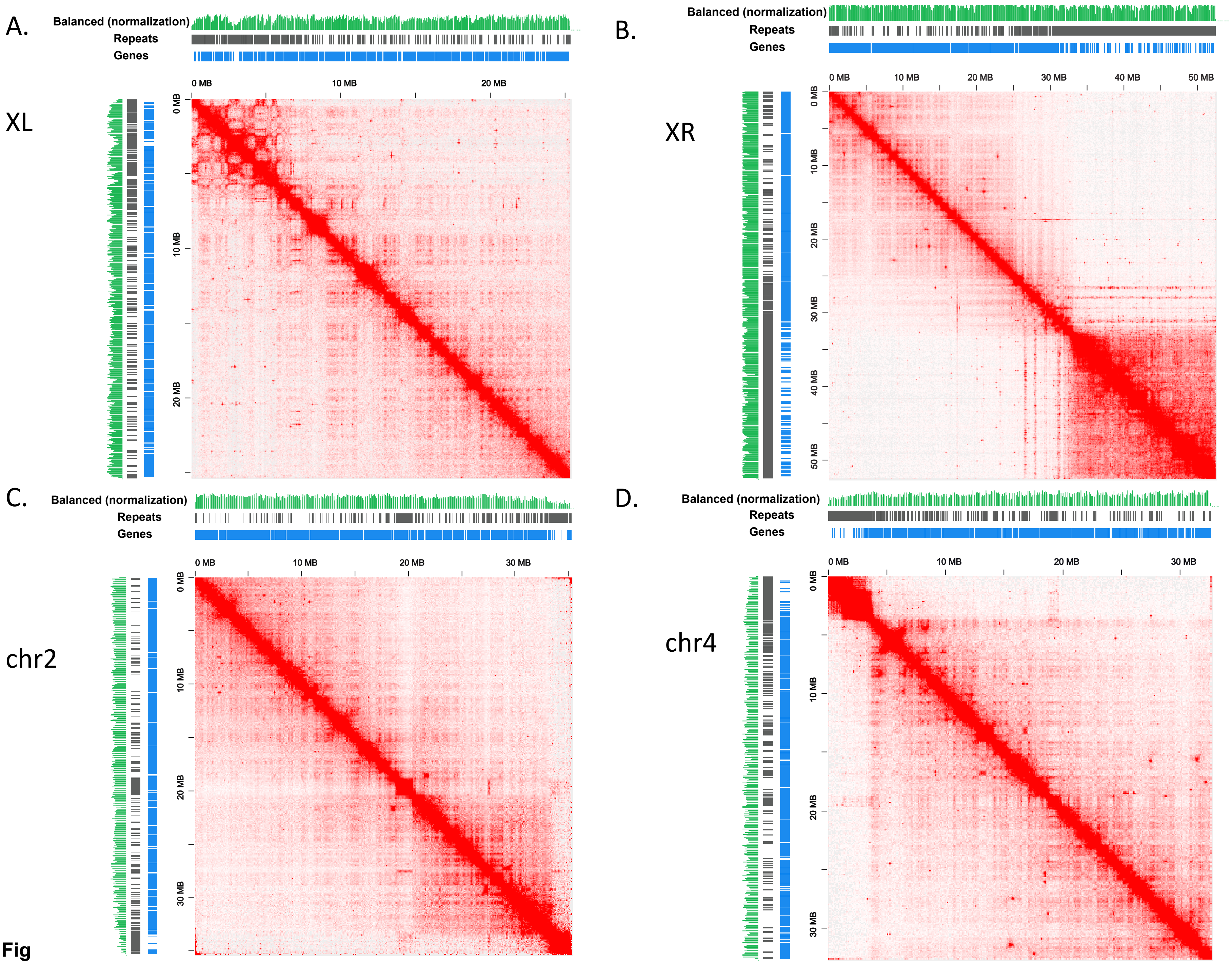
A.
Balanced (normalization)
Repeats
Genes
XL
0 MB
10 MB
20 MB
B.
Balanced (normalization)
Repeats
Genes
XR
0 MB
10 MB
20 MB
30 MB
40 MB
50 MB
C.
Balanced (normalization)
Repeats
Genes
chr2
0 MB
10 MB
20 MB
30 MB
D.
Balanced (normalization)
Repeats
Genes
chr4
0 MB
10 MB
20 MB
30 MB

S3 Fig

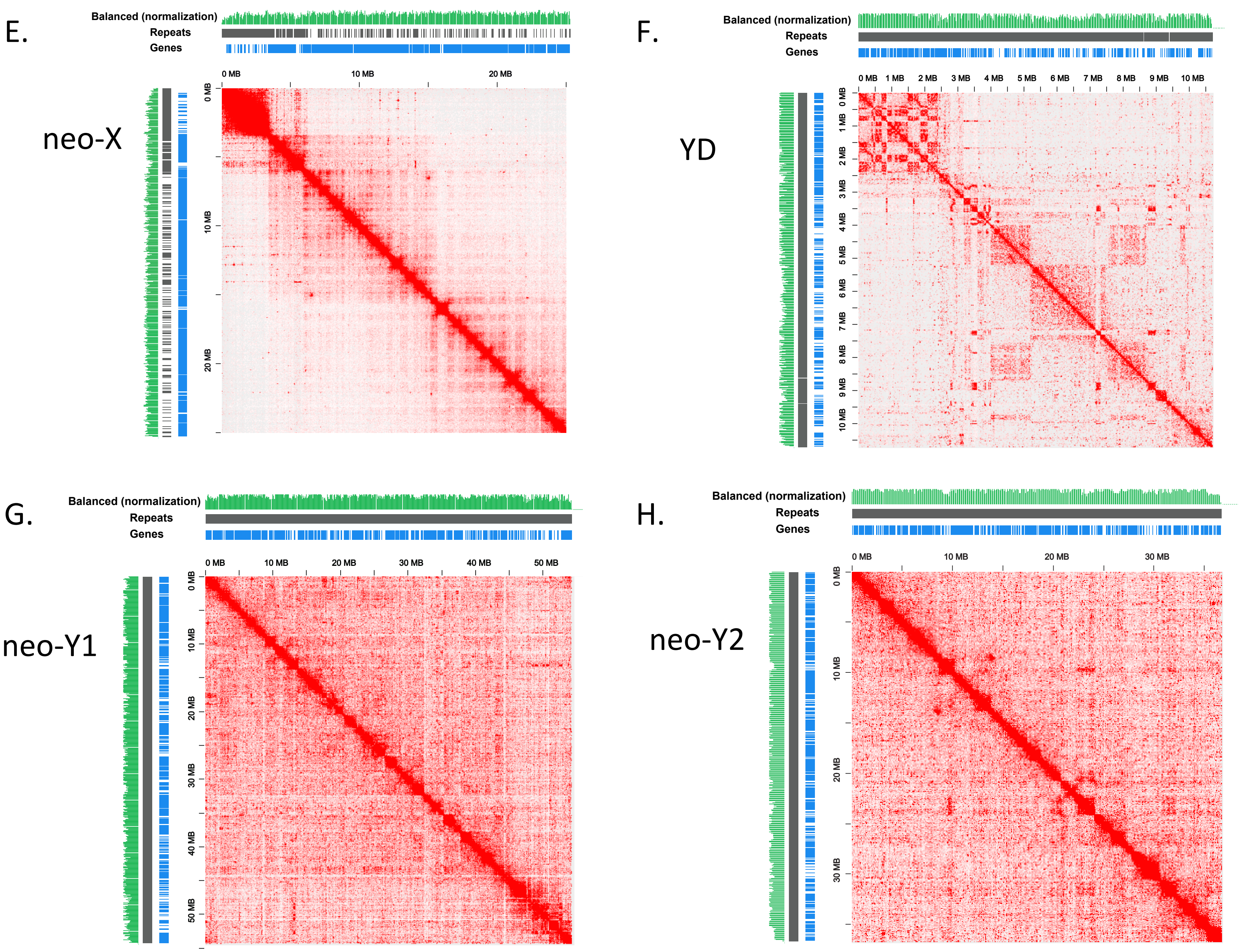
E.
Balanced (normalization)
Repeats
Genes
neo-X
0 MB
10 MB
20 MB
F.
YD
0 MB
1 MB
2 MB
3 MB
4 MB
5 MB
6 MB
7 MB
8 MB
9 MB
10 MB
G.
neo-Y1
0 MB
10 MB
20 MB
30 MB
40 MB
50 MB
H.
neo-Y2
0 MB
10 MB
20 MB
30 MB

S3 Fig

I.

dot

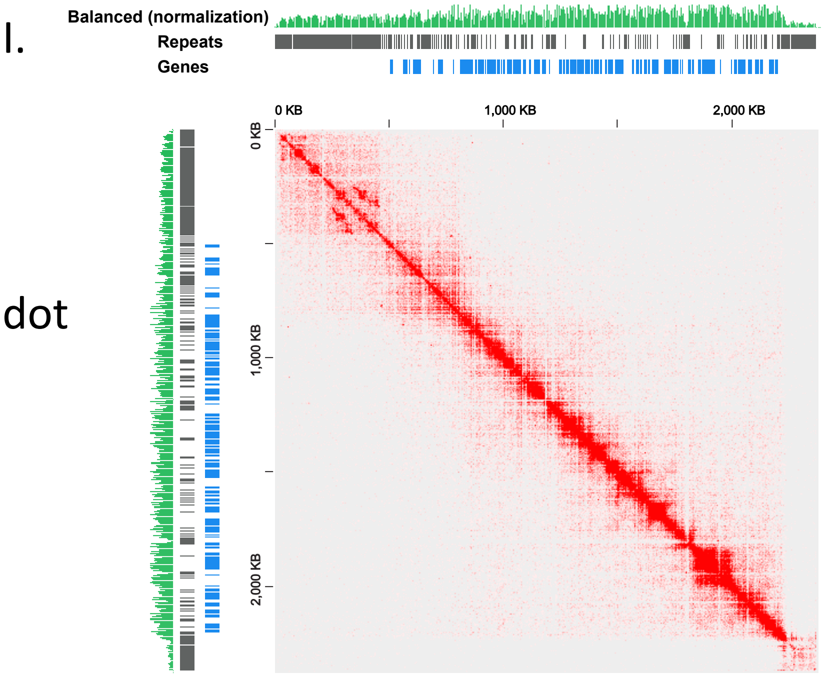

S3 Fig – Hi-C linkage density map, gene and repeat content for **A.** chromosome XL, **B.** chromosome XR, **C.** chromosome 2, **D.** chromosome 4, **E.** neo-X chromosome, **F.** chromosome YD, **G.** neo-Y_1, **H.** neo-Y_2, **I.** Muller F. Neo-Y_1 and neo-Y_2 refer to the two largest neo-Y scaffolds (see Figure 2C). Note that regions of increase repeat density (such as centromeres or the repeat islands on chromosome 2 and 4) show increased contact probabilities with other repeats. Chromosome arms / scaffolds are not drawn to scale.
