# Supplementary material for: De novo assembly of a young Drosophila Y chromosome using single-molecule sequencing and chromatin conformation capture
Source: PLoS Biol. 2018 Jul 30;16(7):e2006348. doi: 10.1371/journal.pbio.2006348 (PMC6117089; doi:10.1371/journal.pbio.2006348)
Supplement: S4 Fig — The normalized female and male Illumina sequence read coverages along the PacBio assembly are plotted in the outer circles in red and blue, respectively. Duplications greater than 100 kb in the assembly are connected with black lines. Erroneously duplicated regions are accompanied by sharp reduction of the coverage by half, because of the sequencing reads being divided between the erroneously duplicated regions (examples marked by arrows). True duplications will show no reduction in read depth. (PDF) [file pbio.2006348.s004.pdf]

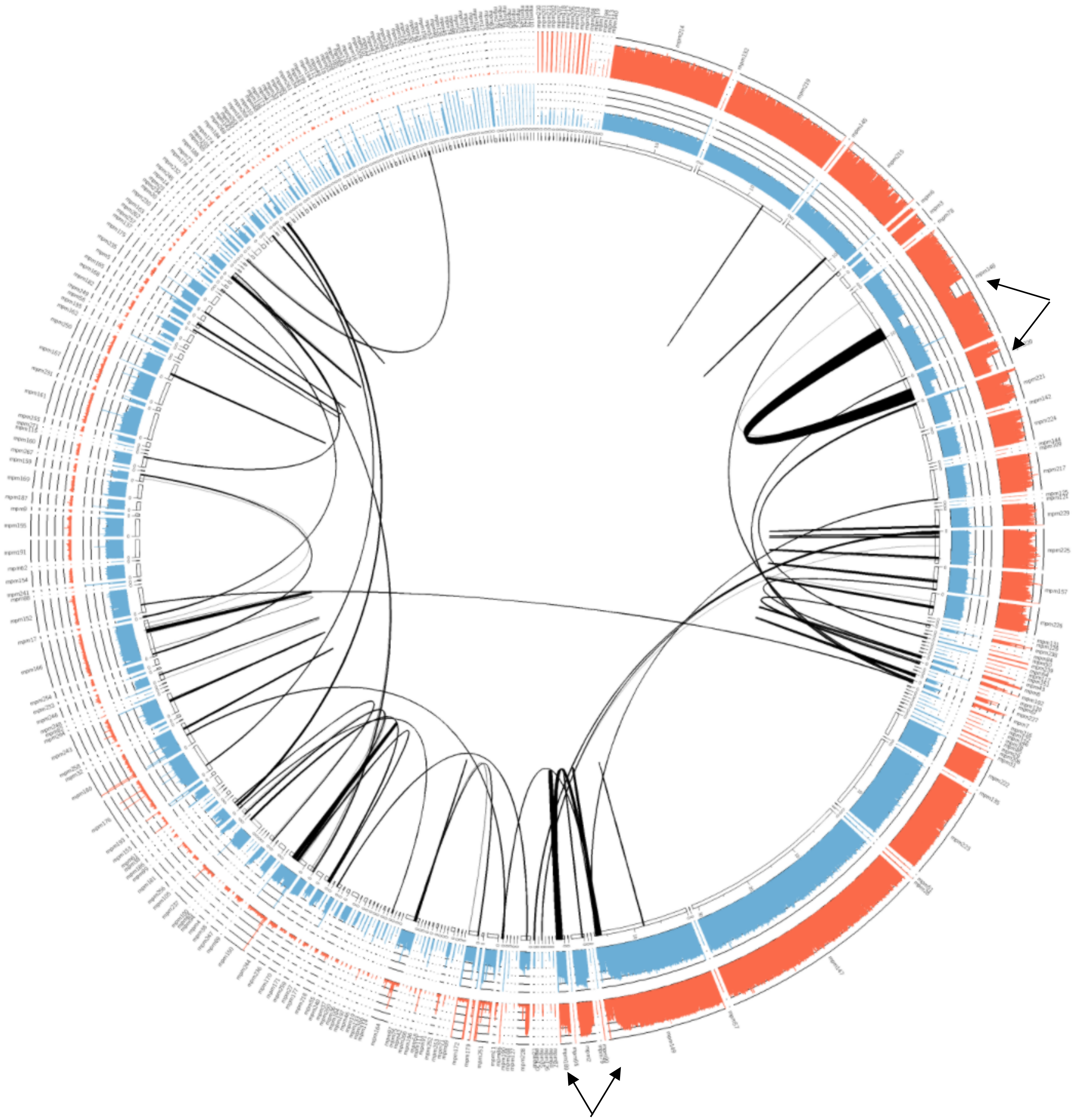

S4 Fig –Erroneous duplications in the PacBio assembly. The normalized female and male Illumina sequence read coverages along the PacBio assembly are plotted in the outer circles in red and blue, respectively. Duplications greater than 100kb in the assembly are connected with black lines. Erroneously duplicated regions are accompanied by sharp reduction of the coverage by half, due to the sequencing reads being divided between the erroneously duplicated regions (examples marked by arrows). True duplications will show no reduction in read depth.
