# Supplementary material for: De novo assembly of a young Drosophila Y chromosome using single-molecule sequencing and chromatin conformation capture
Source: PLoS Biol. 2018 Jul 30;16(7):e2006348. doi: 10.1371/journal.pbio.2006348 (PMC6117089; doi:10.1371/journal.pbio.2006348)
Supplement: S5 Fig — Shown are alignments of BioNano contigs and NGS scaffolds (PacBio and Hi-C scaffolds) to hybrid scaffolds, and alignments of BioNano molecules to HybridScaffolds for the different chromosomes. Chromosome arms/scaffolds are not drawn to scale. NGS, next-generation sequencing. (PDF) [file pbio.2006348.s005.pdf]

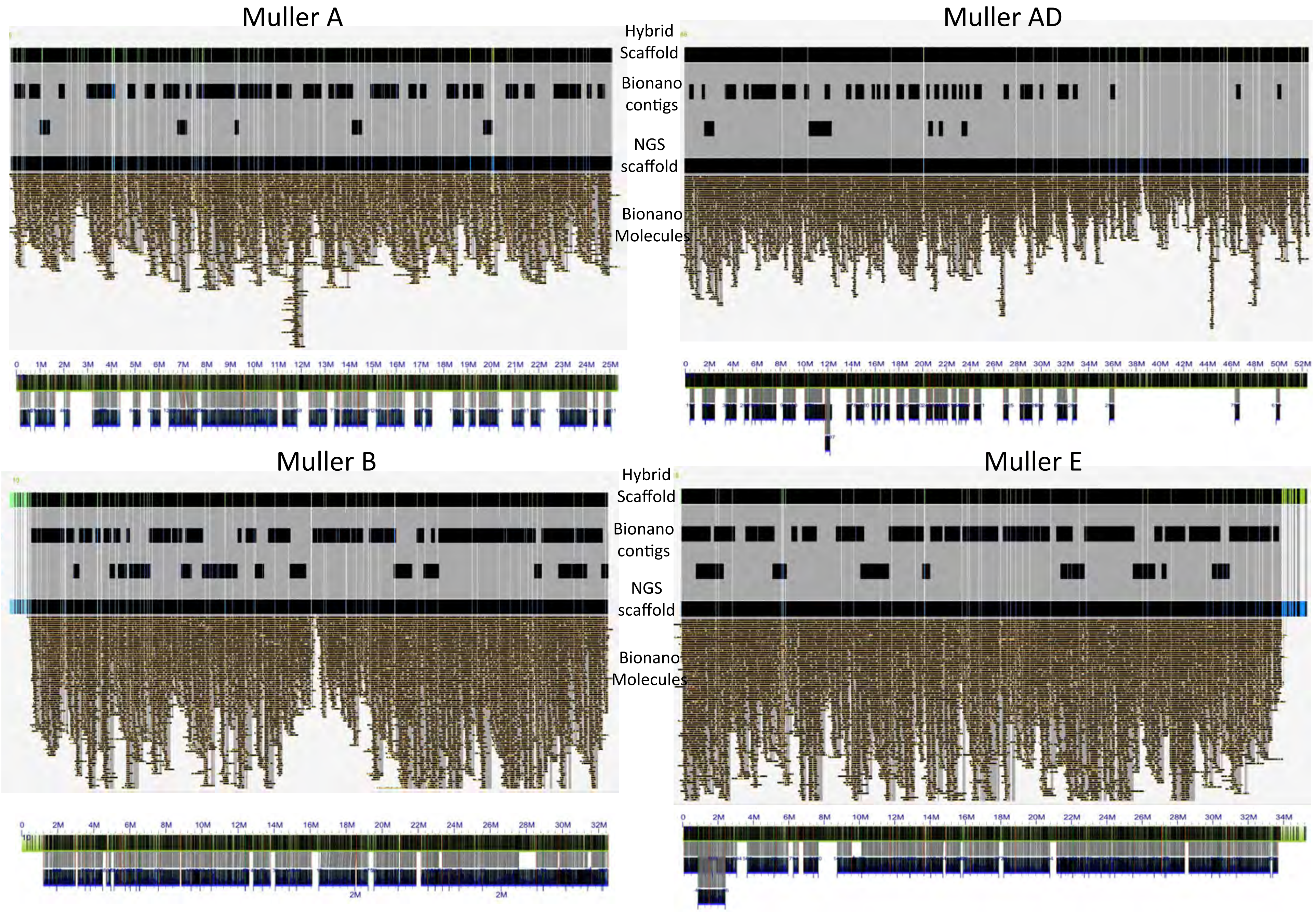
Muller A
Muller AD
Hybrid Scaffold
Bionano contigs
NGS scaffold
Bionano Molecules
0 1M 2M 3M 4M 5M 6M 7M 8M 9M 10M 11M 12M 13M 14M 15M 16M 17M 18M 19M 20M 21M 22M 23M 24M 25M
0 2M 4M 6M 8M 10M 12M 14M 16M 18M 20M 22M 24M 26M 28M 30M 32M 34M 36M 38M 40M 42M 44M 46M 48M 50M 52M
Muller B
Muller E
Hybrid Scaffold
Bionano contigs
NGS scaffold
Bionano Molecules
0 2M 4M 6M 8M 10M 12M 14M 16M 18M 20M 22M 24M 26M 28M 30M 32M
2M
2M
0 2M 4M 6M 8M 10M 12M 14M 16M 18M 20M 22M 24M 26M 28M 30M 32M 34M

S5 Fig

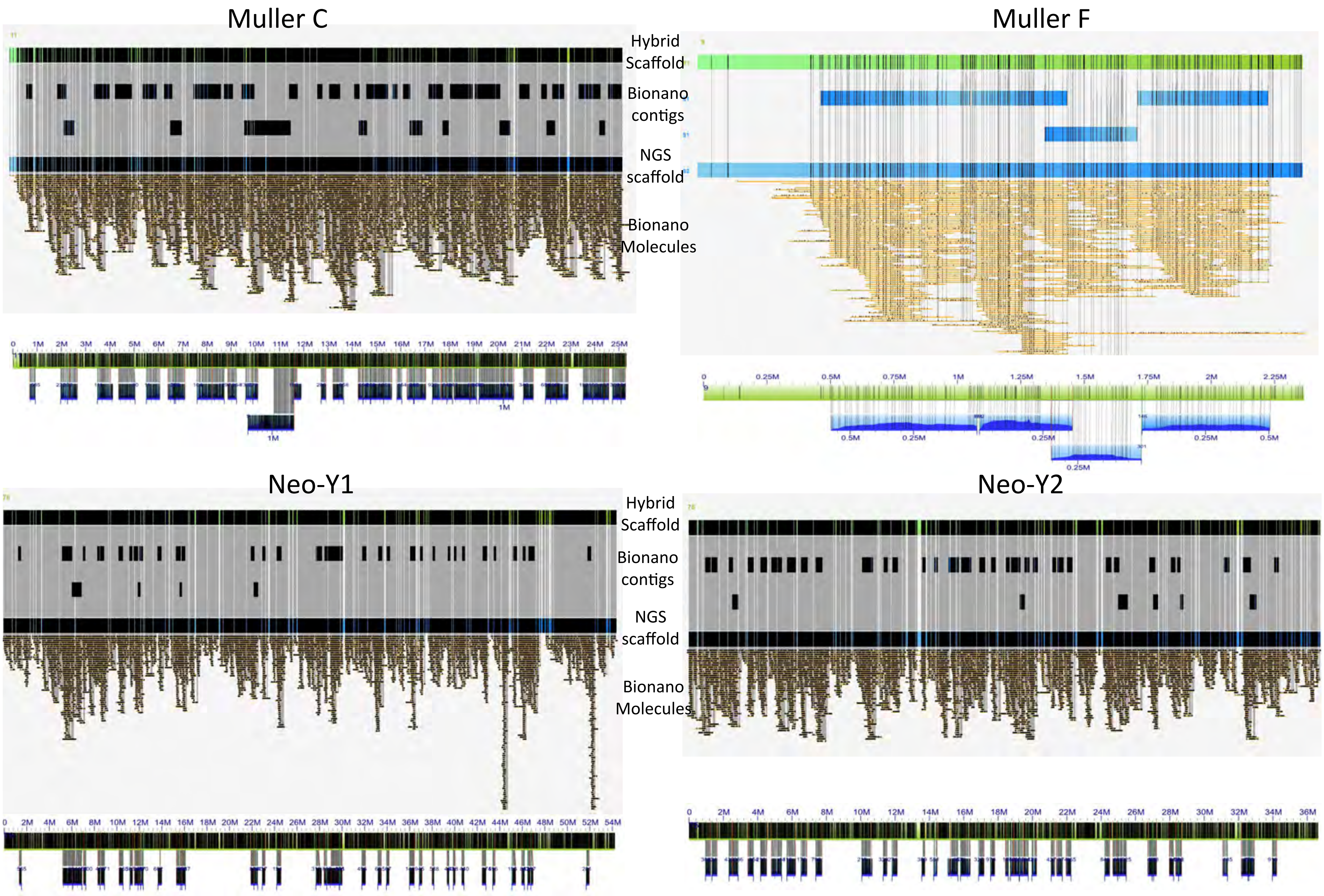

S5 Fig – Validation of our assembly using Bionano optical maps. Shown are alignments of Bionano contigs and NGS scaffolds (PacBio-and Hi-C scaffolds) to hybrid scaffolds, and alignments of Bionano molecules to Hybridscaffolds for the different chromosomes. Chromosome arms / scaffolds are not drawn to scale.
