# Supplementary material for: De novo assembly of a young Drosophila Y chromosome using single-molecule sequencing and chromatin conformation capture
Source: PLoS Biol. 2018 Jul 30;16(7):e2006348. doi: 10.1371/journal.pbio.2006348 (PMC6117089; doi:10.1371/journal.pbio.2006348)
Supplement: S6 Fig — We evaluated IGV plots for all sequenced BAC clones to confirm that they map contiguously and uniquely, and we identified reads mapping to the edge of BAC clones (indicated by red reads). BAC, bacterial artificial chromosome; IGV, integrative genomics viewer. (PDF) [file pbio.2006348.s006.pdf]

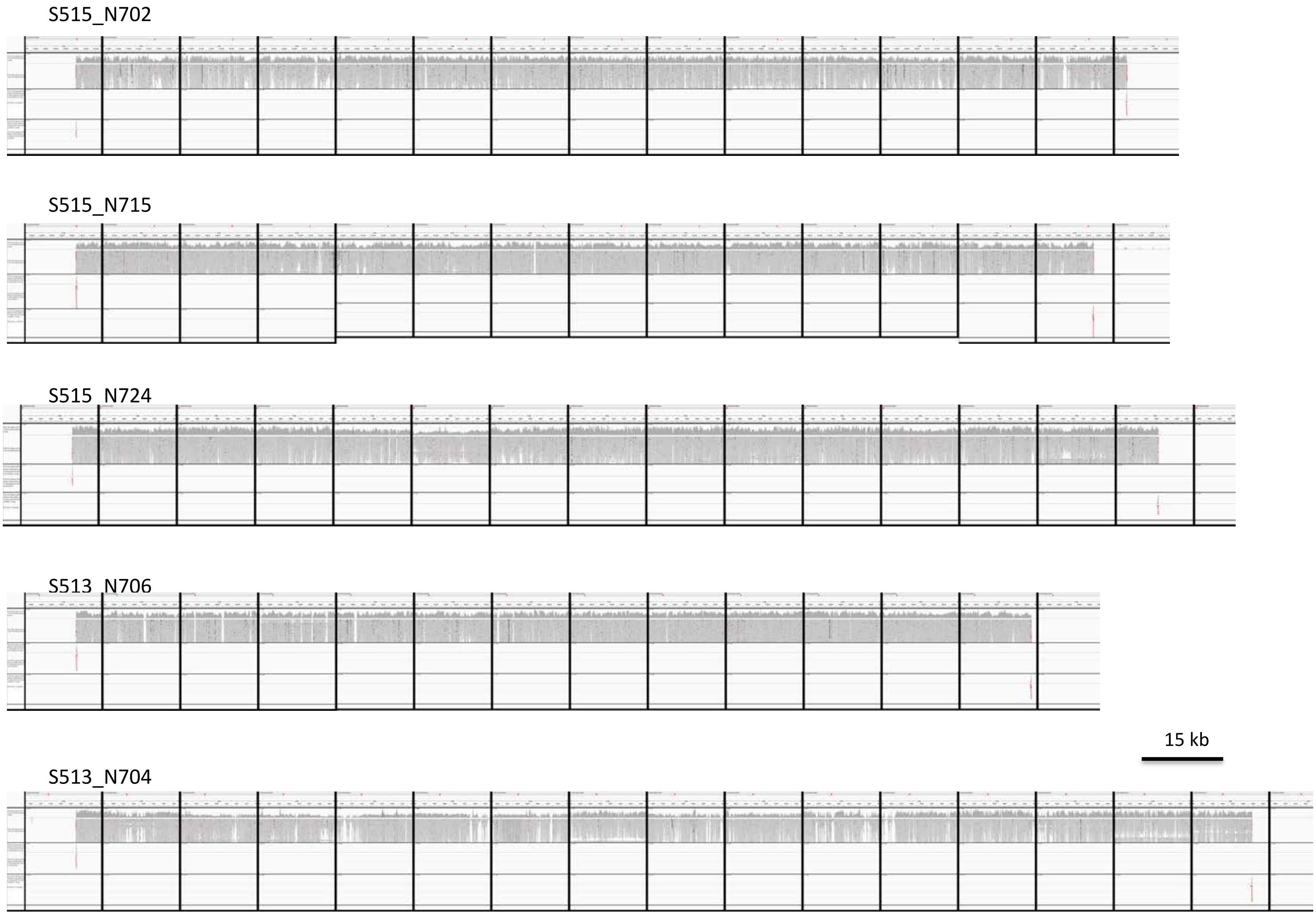

S6 Fig –Mapping of some BAC clones. We evaluated IGV plots for all sequenced BAC clones, to confirm that they map contiguously and uniquely, and we identified reads mapping to the edge of BAC clones (indicated by red reads).
