# Supplementary material for: De novo assembly of a young Drosophila Y chromosome using single-molecule sequencing and chromatin conformation capture
Source: PLoS Biol. 2018 Jul 30;16(7):e2006348. doi: 10.1371/journal.pbio.2006348 (PMC6117089; doi:10.1371/journal.pbio.2006348)
Supplement: S7 Fig — Comparison of current (Dmir2.0) versus old (Dmir1.0) D. miranda assembly for A. chromosome XL, B. chromosome XR, C. chromosome 2, D. chromosome 4, and E. neo-X chromosome. Note that Dmir1.0 contains dozens of inversions that were probably introduced by scaffolding contigs with the D. pseudoobscura genome assembly. Also, Dmir1.0 is substantially shorter, mainly because of the almost complete absence of repetitive sequences from this assembly, such as pericentromeres. (PDF) [file pbio.2006348.s007.pdf]

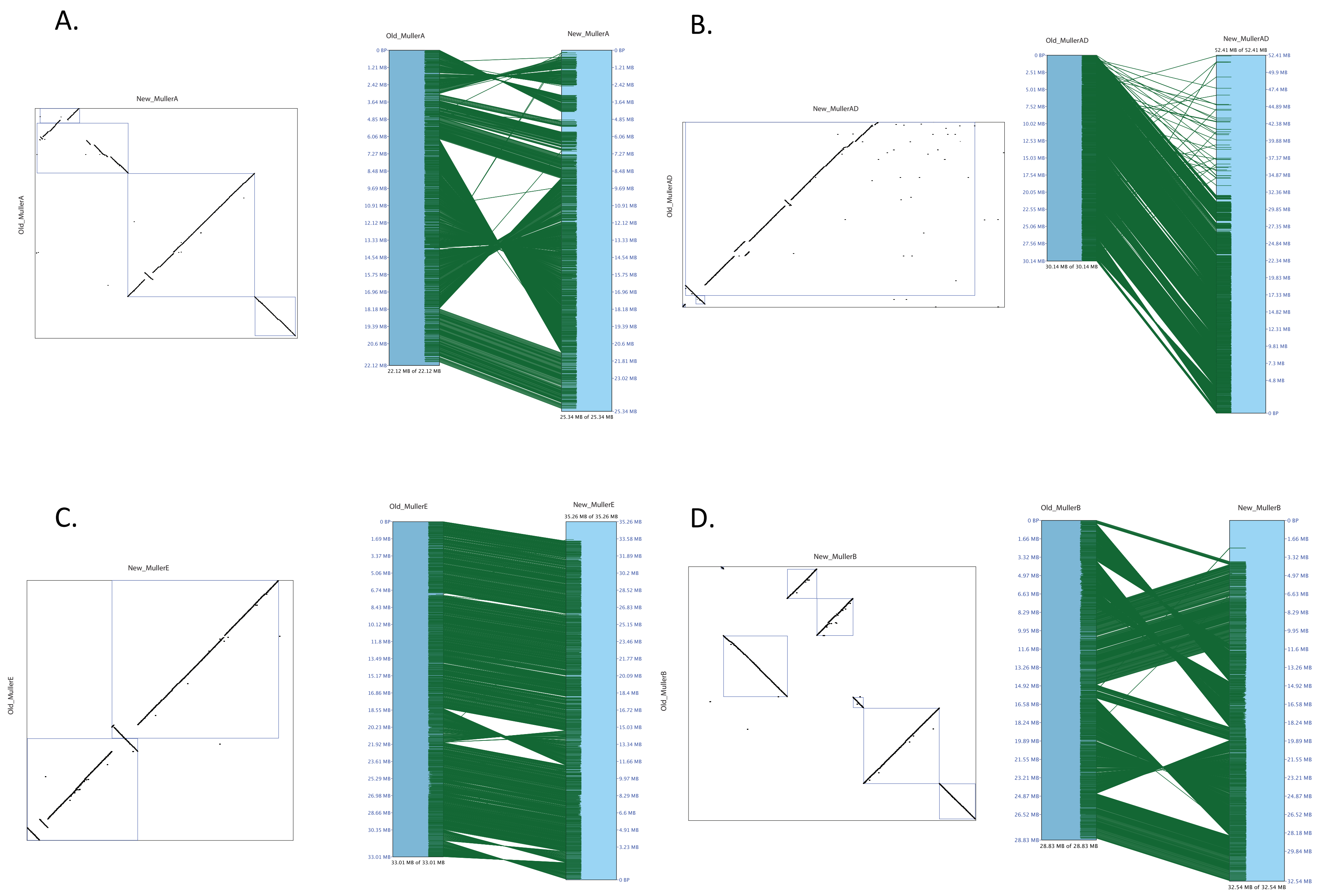
A.
New_MullerA
Old_MullerA
Old_MullerA
New_MullerA
0 BP
1.21 MB
2.42 MB
3.64 MB
4.85 MB
6.06 MB
7.27 MB
8.48 MB
9.69 MB
10.91 MB
12.12 MB
13.33 MB
14.54 MB
15.75 MB
16.96 MB
18.18 MB
19.39 MB
20.6 MB
22.12 MB
22.12 MB of 22.12 MB
21.81 MB
23.02 MB
25.34 MB
25.34 MB of 25.34 MB
B.
New_MullerAD
Old_MullerAD
Old_MullerAD
New_MullerAD
52.41 MB of 52.41 MB
0 BP
2.51 MB
5.01 MB
7.52 MB
10.02 MB
12.53 MB
15.03 MB
17.54 MB
20.05 MB
22.55 MB
25.06 MB
27.56 MB
30.14 MB
30.14 MB of 30.14 MB
52.41 MB
49.9 MB
47.4 MB
44.89 MB
42.38 MB
39.88 MB
37.37 MB
34.87 MB
32.36 MB
29.85 MB
27.35 MB
24.84 MB
22.34 MB
19.83 MB
17.33 MB
14.82 MB
12.31 MB
9.81 MB
7.3 MB
4.8 MB
0 BP
C.
New_MullerE
Old_MullerE
Old_MullerE
New_MullerE
35.26 MB of 35.26 MB
0 BP
1.69 MB
3.37 MB
5.06 MB
6.74 MB
8.43 MB
10.12 MB
11.8 MB
13.49 MB
15.17 MB
16.86 MB
18.55 MB
20.23 MB
21.92 MB
23.61 MB
25.29 MB
26.98 MB
28.66 MB
30.35 MB
33.01 MB
33.01 MB of 33.01 MB
35.26 MB
33.58 MB
31.89 MB
30.2 MB
28.52 MB
26.83 MB
25.15 MB
23.46 MB
21.77 MB
20.09 MB
18.4 MB
16.72 MB
15.03 MB
13.34 MB
11.66 MB
9.97 MB
8.29 MB
6.6 MB
4.91 MB
3.23 MB
0 BP
D.
New_MullerB
Old_MullerB
Old_MullerB
New_MullerB
0 BP
1.66 MB
3.32 MB
4.97 MB
6.63 MB
8.29 MB
9.95 MB
11.6 MB
13.26 MB
14.92 MB
16.58 MB
18.24 MB
19.89 MB
21.55 MB
23.21 MB
24.87 MB
26.52 MB
28.83 MB
28.83 MB of 28.83 MB
28.18 MB
29.84 MB
32.54 MB
32.54 MB of 32.54 MB

S7 Fig

E.

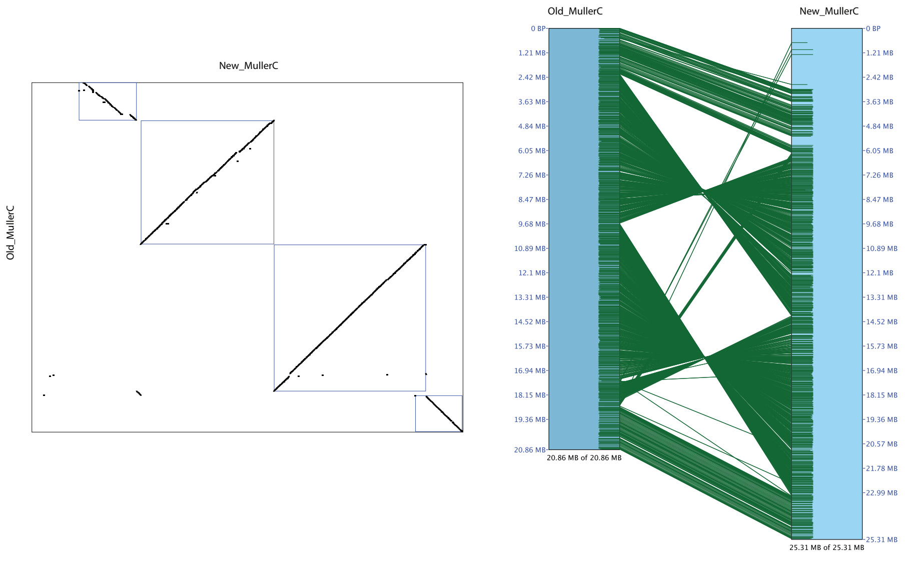

S7 Fig – Comparison of current (Dmir2.0) vs. old (Dmir1.0) *D. miranda* assembly for **A.** chromosome XL, **B.** chromosome XR, **C.** chromosome 2, **D.** chromosome 4, **E.** neo-X chromosome. Note that Dmir1.0 contains dozens of inversions that were probably introduced by scaffolding contigs with the *D. pseudoobscura* genome assembly. Also, Dmir1.0 is substantially shorter, mainly due to the almost complete absence of repetitive sequences from this assembly, such as pericentromeres.
