# Supplementary figures and images for: De novo assembly of a young Drosophila Y chromosome using single-molecule sequencing and chromatin conformation capture
Source: PLoS Biol. 2018 Jul 30;16(7):e2006348. doi: 10.1371/journal.pbio.2006348 (PMC6117089; doi:10.1371/journal.pbio.2006348)

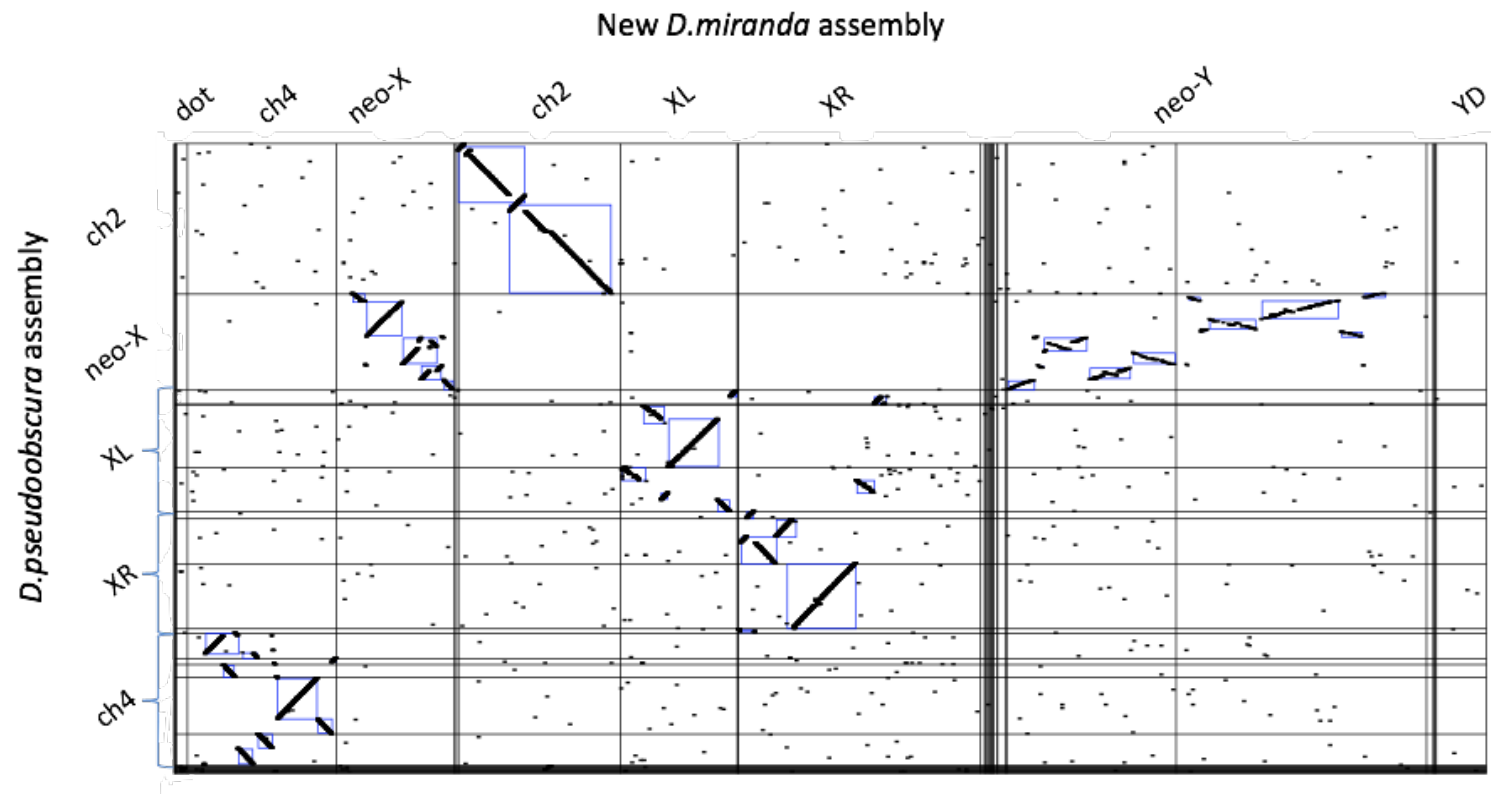

**S8 Fig** – Comparison of current *D. miranda* assembly (Dmir2.0) vs. *D. pseudoobscura* assembly.

Supplement: S8 Fig — (PDF) [file pbio.2006348.s008.pdf]
