# Supplementary material for: De novo assembly of a young Drosophila Y chromosome using single-molecule sequencing and chromatin conformation capture
Source: PLoS Biol. 2018 Jul 30;16(7):e2006348. doi: 10.1371/journal.pbio.2006348 (PMC6117089; doi:10.1371/journal.pbio.2006348)

A.

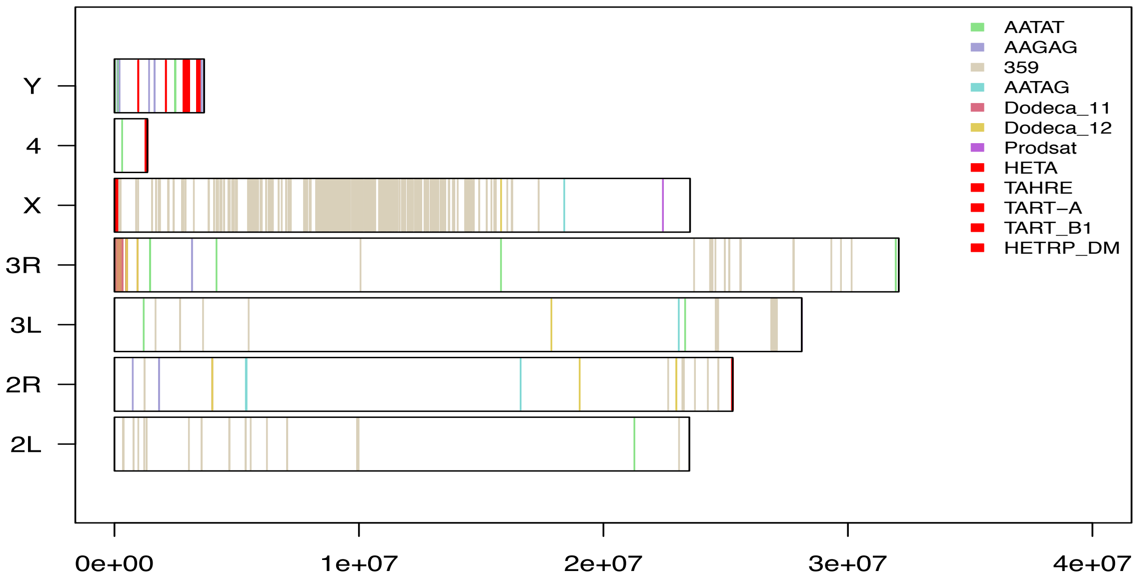

S9 Fig – Satellite DNA in the *D. melanogaster* assembly (r6). **A.** Distribution of centromeric satellites and telomeric retrotransposons (color labeled) are plotted along the scaffolds. **B.** The first (left) and last (right) 1Mb are plotted for each chromosome arm. Note that while the *D. melanogaster* chromosome arms typically have their telomeres assembled (red repeats), centromeric repeats (all other repeats) are generally missing from the assembly.

B.

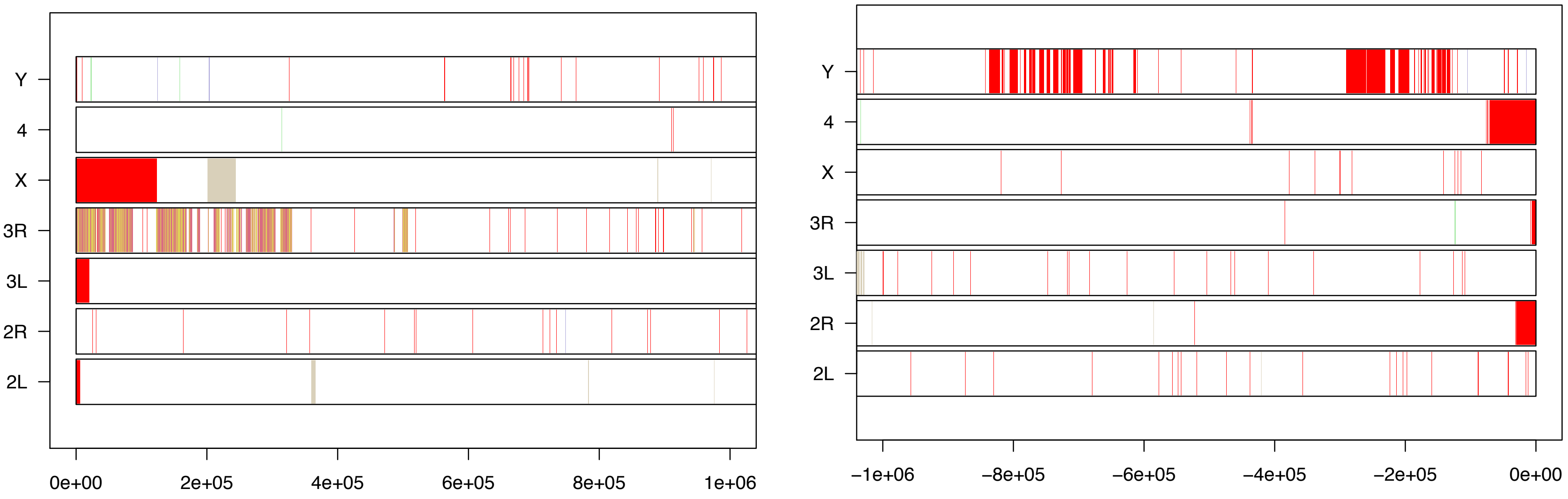

Supplement: S9 Fig — A. Distribution of centromeric satellites and telomeric retrotransposons (color labeled) are plotted along the scaffolds. B. The first (left) and last (right) 1 Mb are plotted for each chromosome arm. Note that while the D. melanogaster chromosome arms typically have their telomeres assembled (red repeats), centromeric repeats (all other repeats) are generally missing from the assembly. (PDF) [file pbio.2006348.s009.pdf]
