# Supplementary material for: De novo assembly of a young Drosophila Y chromosome using single-molecule sequencing and chromatin conformation capture
Source: PLoS Biol. 2018 Jul 30;16(7):e2006348. doi: 10.1371/journal.pbio.2006348 (PMC6117089; doi:10.1371/journal.pbio.2006348)
Supplement: S10 Fig — Many tandemly duplicated regions were poorly represented in the published assembly and generally collapsed into a single copy. (PDF) [file pbio.2006348.s010.pdf]

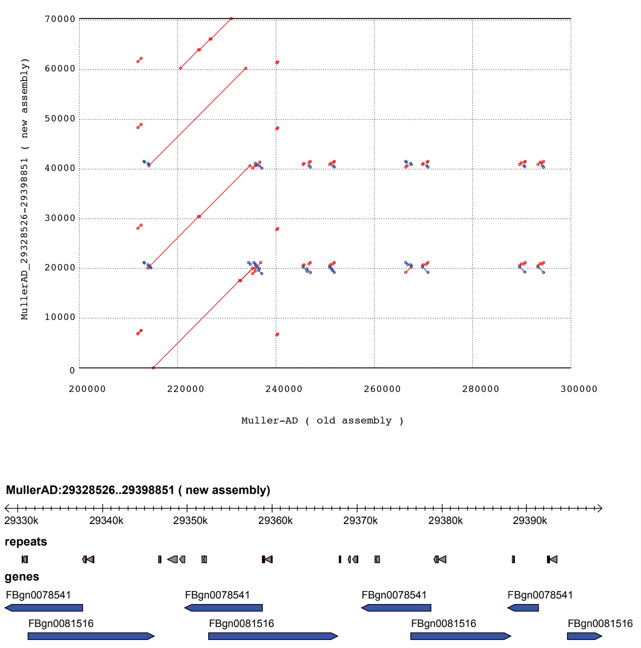

MullerAD_29328526-29398851 (new assembly)
Muller-AD (old assembly)
MullerAD:29328526..29398851 (new assembly)
29330k
29340k
29350k
29360k
29370k
29380k
29390k
repeats
genes
FBgn0078541
FBgn0078541
FBgn0078541
FBgn0078541
FBgn0081516
FBgn0081516
FBgn0081516
FBgn0081516

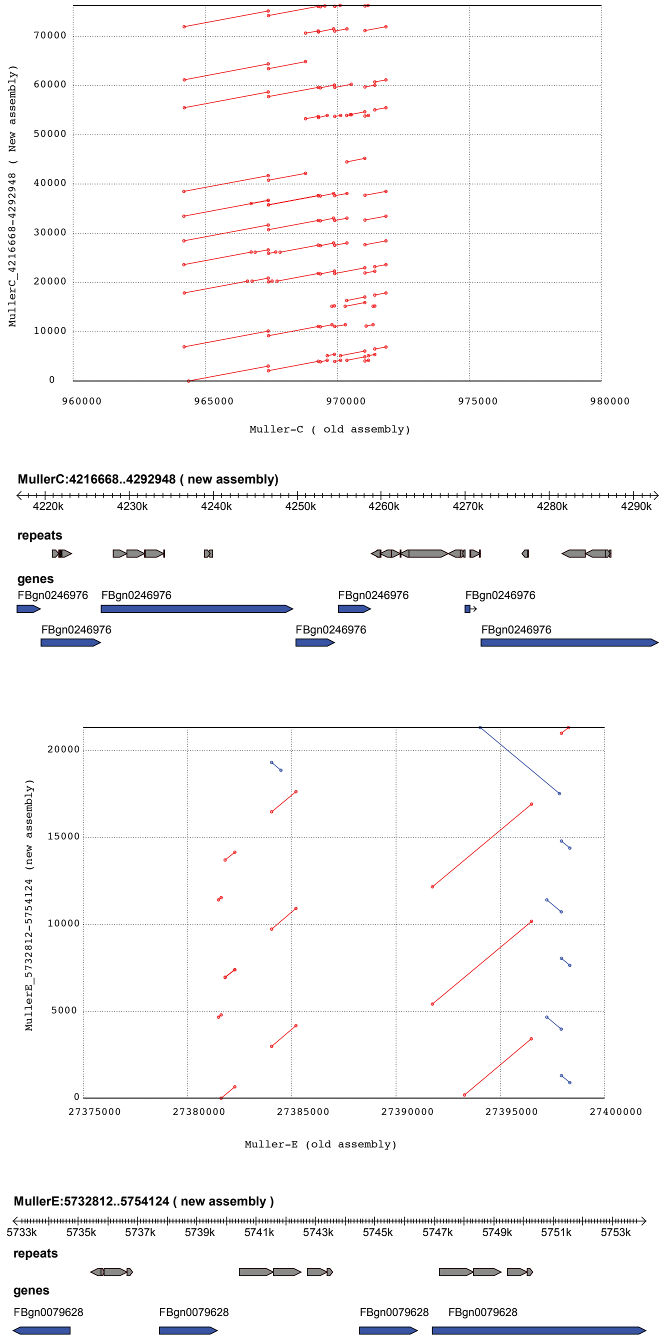

MullerC_4216668-4292948 (New assembly)
Muller-C (old assembly)
MullerC:4216668..4292948 (new assembly)
4220k
4230k
4240k
4250k
4260k
4270k
4280k
4290k
repeats
genes
FBgn0246976
FBgn0246976
FBgn0246976
FBgn0246976
FBgn0246976
FBgn0246976
FBgn0246976
MullerE_5732812-5754124 (new assembly)
Muller-E (old assembly)
MullerE:5732812..5754124 (new assembly)
5733k
5735k
5737k
5739k
5741k
5743k
5745k
5747k
5749k
5751k
5753k
repeats
genes
FBgn0079628
FBgn0079628
FBgn0079628
FBgn0079628

S10 Fig

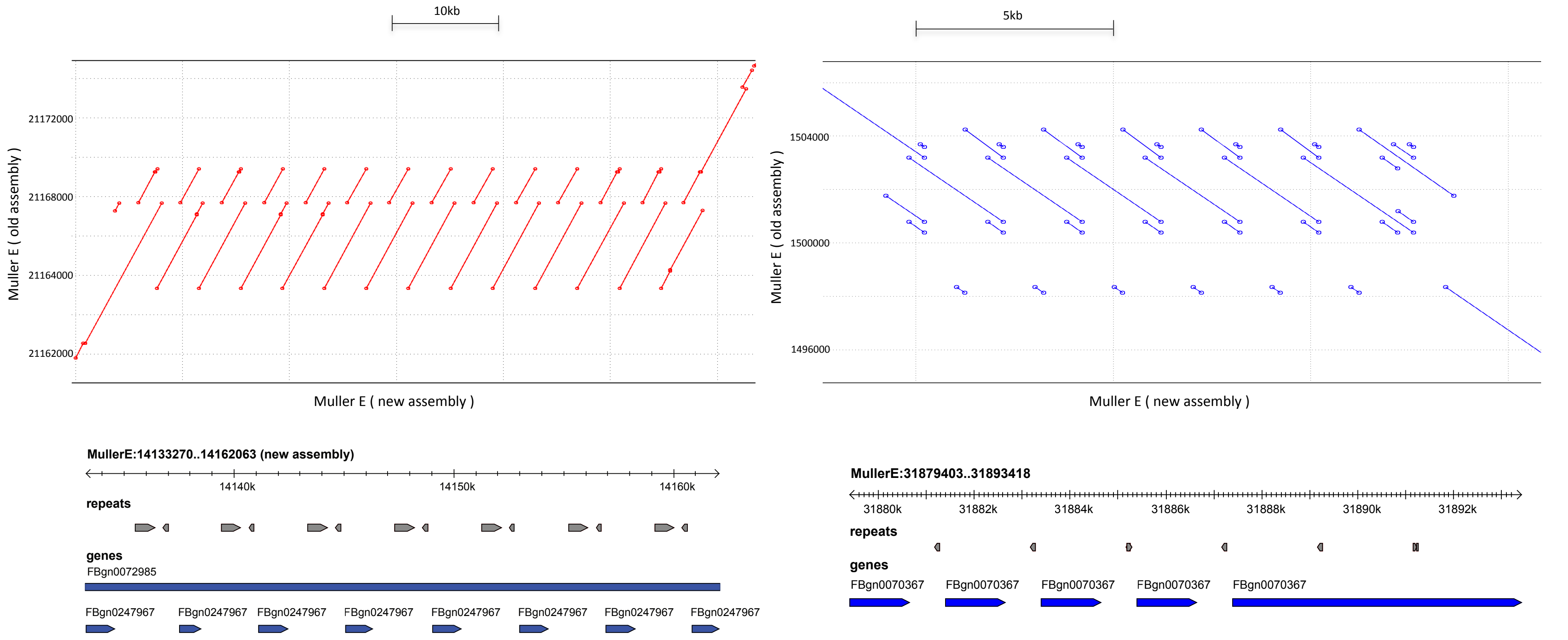

S10 Fig – Comparative alignments of resolved tandemly duplicated gene clusters in *D. miranda*. Many tandemly duplicated regions were poorly represented in the published assembly and generally collapsed into a single copy.
