# Supplementary material for: De novo assembly of a young Drosophila Y chromosome using single-molecule sequencing and chromatin conformation capture
Source: PLoS Biol. 2018 Jul 30;16(7):e2006348. doi: 10.1371/journal.pbio.2006348 (PMC6117089; doi:10.1371/journal.pbio.2006348)
Supplement: S11 Fig — BAC clone S506-N718 on Muller E is located in the middle of a 21-bp repeat region, supporting that our assembly is of high quality in the repeat-rich centromere and pericentromeric regions. BAC, bacterial artificial chromosome. (PDF) [file pbio.2006348.s011.pdf]

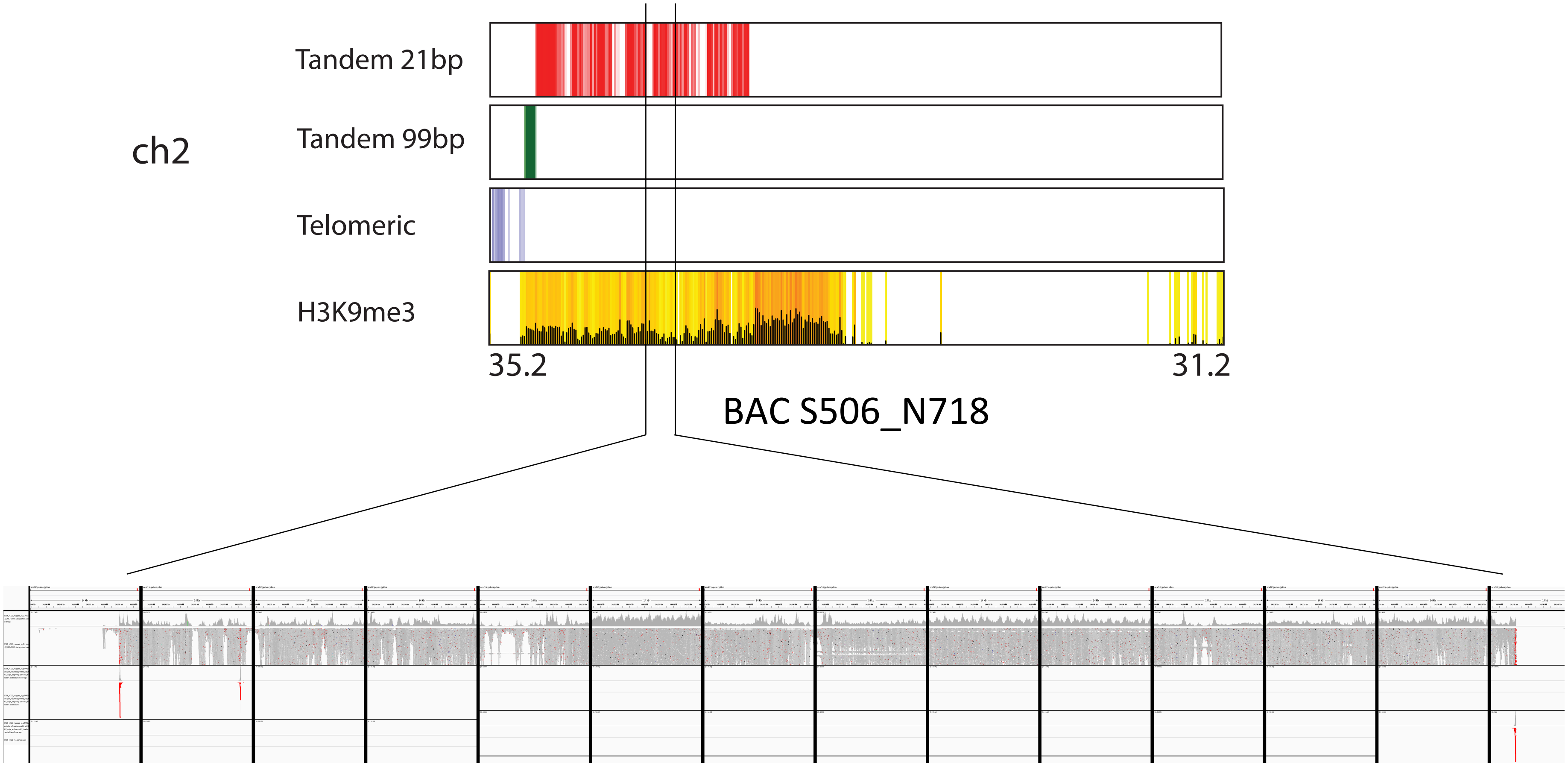

S11 Fig – BAC clone sequencing confirms centromere assembly. BAC clone S506-N718 on Muller E is located in the middle of a 21-bp repeat region, supporting that our assembly is of high quality in the repeat-rich centromere and pericentromeric regions.
