# Supplementary material for: De novo assembly of a young Drosophila Y chromosome using single-molecule sequencing and chromatin conformation capture
Source: PLoS Biol. 2018 Jul 30;16(7):e2006348. doi: 10.1371/journal.pbio.2006348 (PMC6117089; doi:10.1371/journal.pbio.2006348)
Supplement: S12 Fig — A. Distribution of satellites and telomeric retrotransposons (color labeled) are plotted along the scaffolds. The names of satellites are derived from the length of the base motif. Note that the 84-bp is a complex structure of four 21-bp variants. B. The first and last 1.5-Mb is plotted for select chromosomes. C. The abundances of each satellite type across the assembly. D. The number of base pairs masked by each satellite type. Underlying data can be found in S1 Data. (PDF) [file pbio.2006348.s012.pdf]

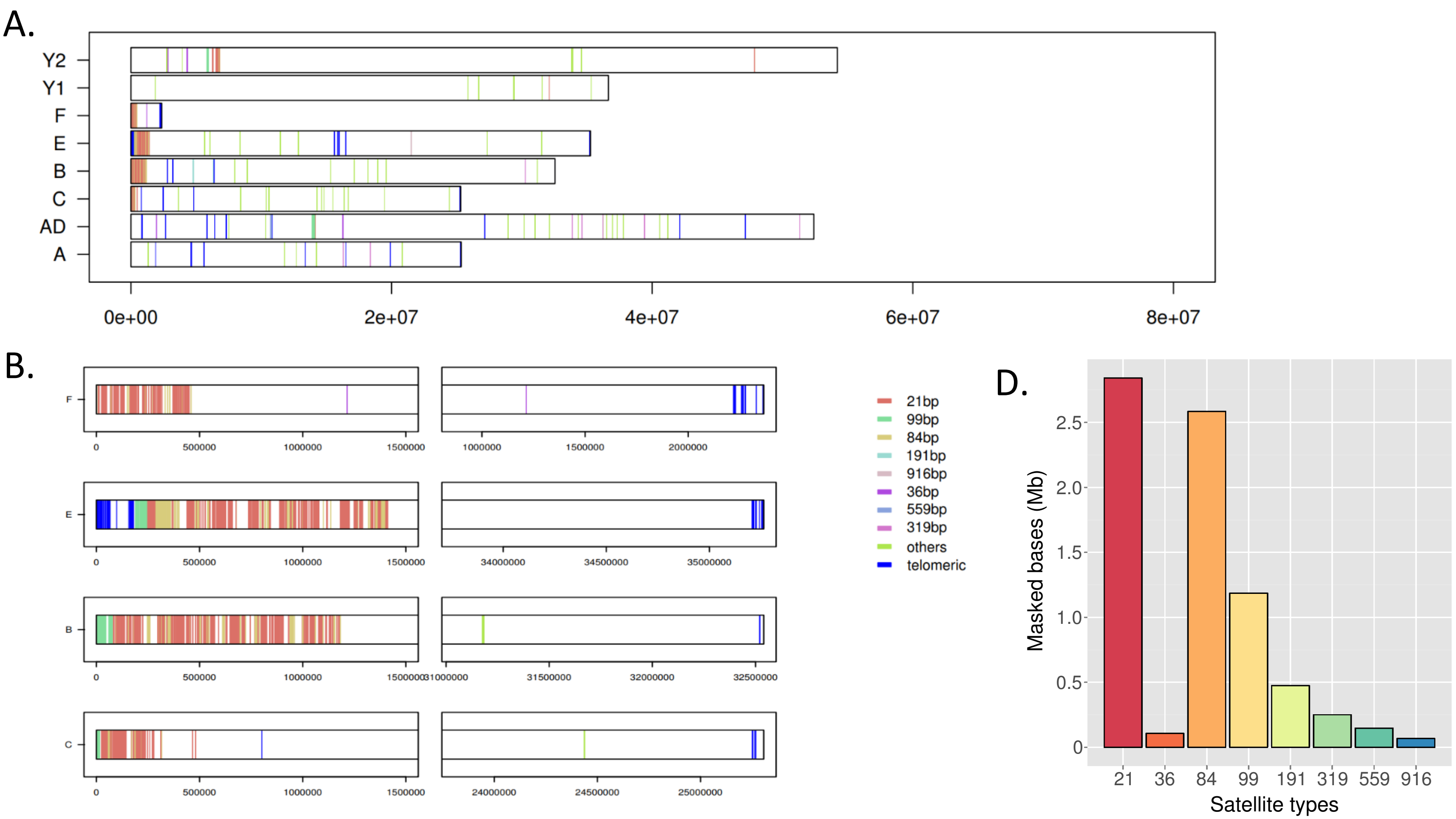

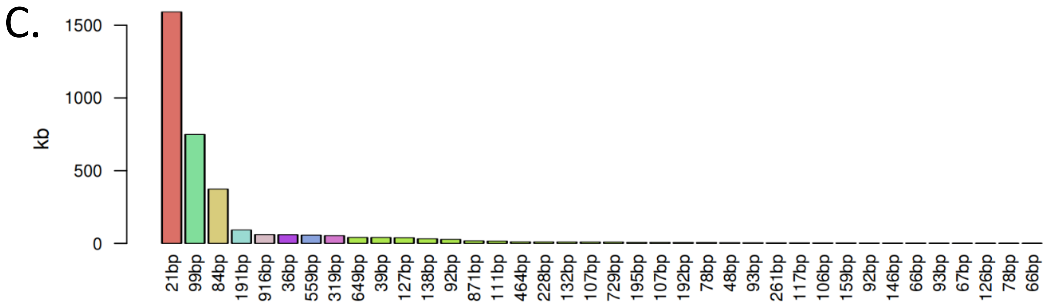

S12 Fig – Satellite DNA in the assembly. **A.** Distribution of satellites and telomeric retrotransposons (color labeled) are plotted along the scaffolds. The name of satellites are derived from the length of the base motif. Note that the 84-bp is a complex structure of four 21-bp variants. **B.** The first and last 1.5 Mb is plotted for select chromosomes. **C.** The abundances of each satellite type across the assembly. **D.** The number of base-pairs masked by each satellite type. Underlying data can be found in S1_Data.xlsx.
