# Supplementary material for: De novo assembly of a young Drosophila Y chromosome using single-molecule sequencing and chromatin conformation capture
Source: PLoS Biol. 2018 Jul 30;16(7):e2006348. doi: 10.1371/journal.pbio.2006348 (PMC6117089; doi:10.1371/journal.pbio.2006348)
Supplement: S13 Fig — Statistical significance was calculated using a Wilcoxon test. Underlying data can be found in S1 Data. H3K9me3, trimethylation of histone 3 lysine 9. (PDF) [file pbio.2006348.s013.pdf]

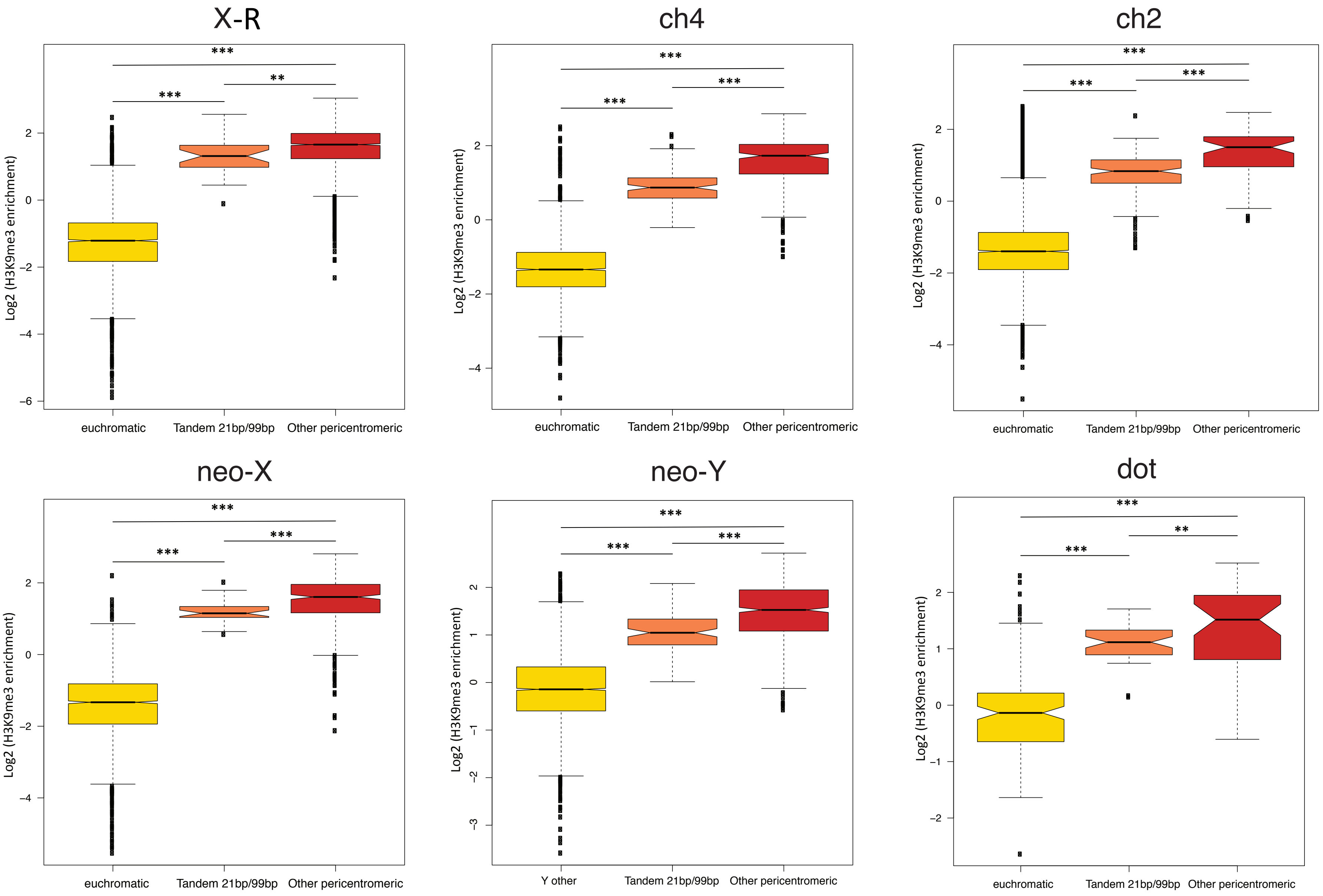

S13 Fig – Enrichment of H3K9me3 at pericentromeric regions and putative centromeric repeat for different chromosome arms (note that X-L and YD show no large regions containing the 21-bp or 99-bp repeat motif and are not shown). Statistical significance was calculated using a Wilcoxon test. Underlying data can be found in S1_Data.xlsx.
