# Supplementary material for: De novo assembly of a young Drosophila Y chromosome using single-molecule sequencing and chromatin conformation capture
Source: PLoS Biol. 2018 Jul 30;16(7):e2006348. doi: 10.1371/journal.pbio.2006348 (PMC6117089; doi:10.1371/journal.pbio.2006348)
Supplement: S14 Fig — A. Shown is mapping of Illumina reads from neo-X-derived BAC clones to their (I) correct neo-X genomic location, (II) to their homologous neo-Y region, and (III) to their homologous neo-Y region after masking the correct neo-X location. We see little cross-mapping of neo-X-derived BAC clone reads to the homologous neo-Y location (see II), and neo-X reads only start mapping to their homologous neo-Y region (with many SNPs, as indicated by the colors in the coverage track) after the neo-X region is masked (see III), revealing their former homology. Little cross-mapping of neo-X reads to the neo-Y chromosome confirms the high quality of our assembly, and lack of chimeric sequences. Also note that the homologous neo-Y segment is considerably larger than the neo-X, because of the accumulation of repetitive sequences on the neo-Y. B. Shown is mapping of Illumina reads from neo-Y-derived BAC clones to their (I) correct neo-Y genomic location, (II) to their homologous neo-X region, and (III) to their homologous neo-X region after masking the correct neo-Y location. We see little cross-mapping of neo-Y-derived BAC clone reads to their homologous neo-X location (see II), and neo-Y reads only start mapping to their homologous neo-X region (with many SNPs, as indicated by the colors in the coverage track) after the neo-Y region is masked (see III), revealing their former homology. Little cross-mapping of neo-Y reads to the neo-X chromosome confirms the high quality of our assembly, and lack of chimeric sequences. Also note that the homologous neo-X segment is considerably smaller than the neo-Y, because of the accumulation of repetitive sequences on the neo-Y. BAC, bacterial artificial chromosome. (PDF) [file pbio.2006348.s014.pdf]

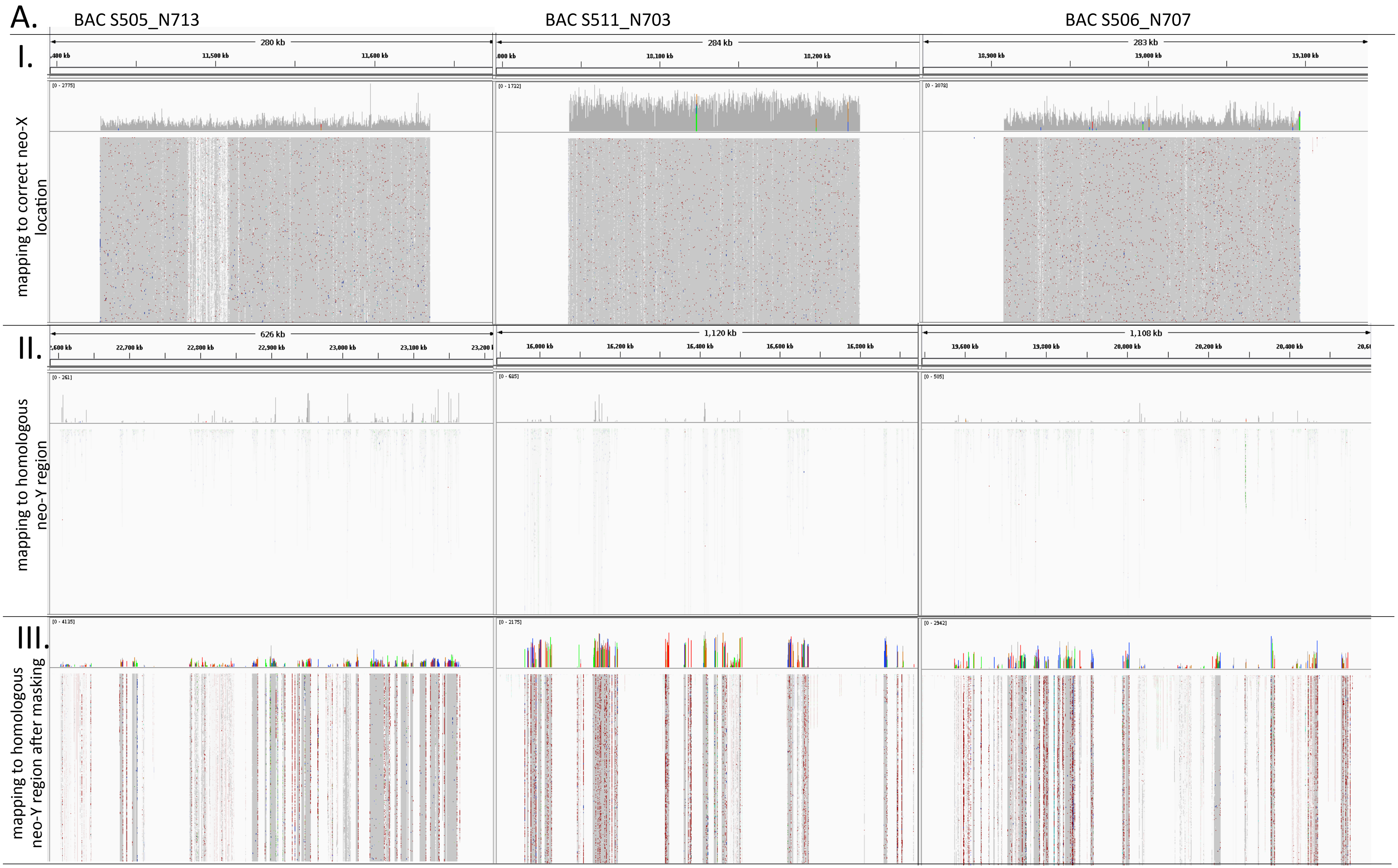

S14 Fig – Validation of lack of chimeric sequence assemblies for neo-X and neo-Y regions. A. Shown is mapping of Illumina reads from neo-X derived BAC clones to their (I.) correct neo-X genomic location, (II.) to their homologous neo-Y region, (III.) to their homologous neo-Y region after masking the correct neo-X location. We see little cross-mapping of neo-X-derived BAC clone reads to the homologous neo-Y location (see II), and neo-X reads only start mapping to their homologous neo-Y region (with many SNPs as indicated by the colors in the coverage track) after the neo-X region is masked (see III), revealing their former homology. Little cross-mapping of neo-X reads to the neo-Y chromosome confirms the high quality of our assembly, and lack of chimeric sequences. Also note that the homologous neo-Y segment is considerably larger than the neo-X, due to the accumulation of repetitive sequences on the neo-Y.

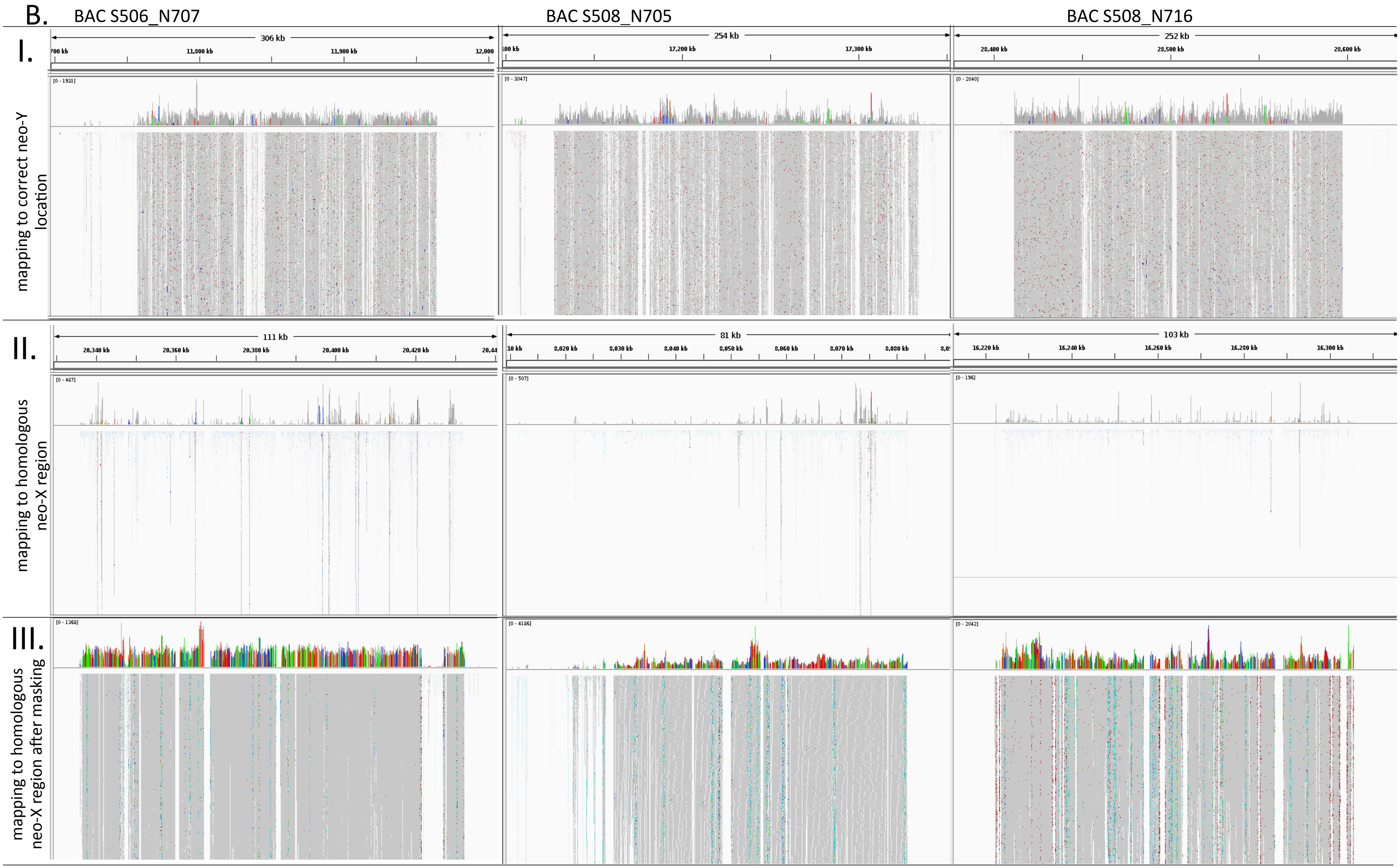

S14 Fig – Validation of lack of chimeric sequence assemblies for neo-X and neo-Y regions. B. Shown is mapping of Illumina reads from neo-Y derived BAC clones to their (I.) correct neo-Y genomic location, (II.) to their homologous neo-X region, (III.) to their homologous neo-X region after masking the correct neo-Y location. We see little cross-mapping of neo-Y-derived BAC clone reads to their homologous neo-X location (see II), and neo-Y reads only start mapping to their homologous neo-X region (with many SNPs as indicated by the colors in the coverage track) after the neo-Y region is masked (see III), revealing their former homology. Little cross-mapping of neo-Y reads to the neo-X chromosome confirms the high quality of our assembly, and lack of chimeric sequences. Also note that the homologous neo-X segment is considerably smaller than the neo-Y, due to the accumulation of repetitive sequences on the neo-Y.
