# Supplementary material for: De novo assembly of a young Drosophila Y chromosome using single-molecule sequencing and chromatin conformation capture
Source: PLoS Biol. 2018 Jul 30;16(7):e2006348. doi: 10.1371/journal.pbio.2006348 (PMC6117089; doi:10.1371/journal.pbio.2006348)
Supplement: S15 Fig — A. Homologous blocks on the neo-X and neo-Y. B. Global alignments between homologous regions on the neo-X and neo-Y. C. Local alignments between homologous neo-X and neo-Y regions chosen at random. The red bars indicate repeats. The scaffolds neo-Y1 and neo-Y2 refer to the two largest neo-Y scaffolds (see Fig 2C). (PDF) [file pbio.2006348.s015.pdf]

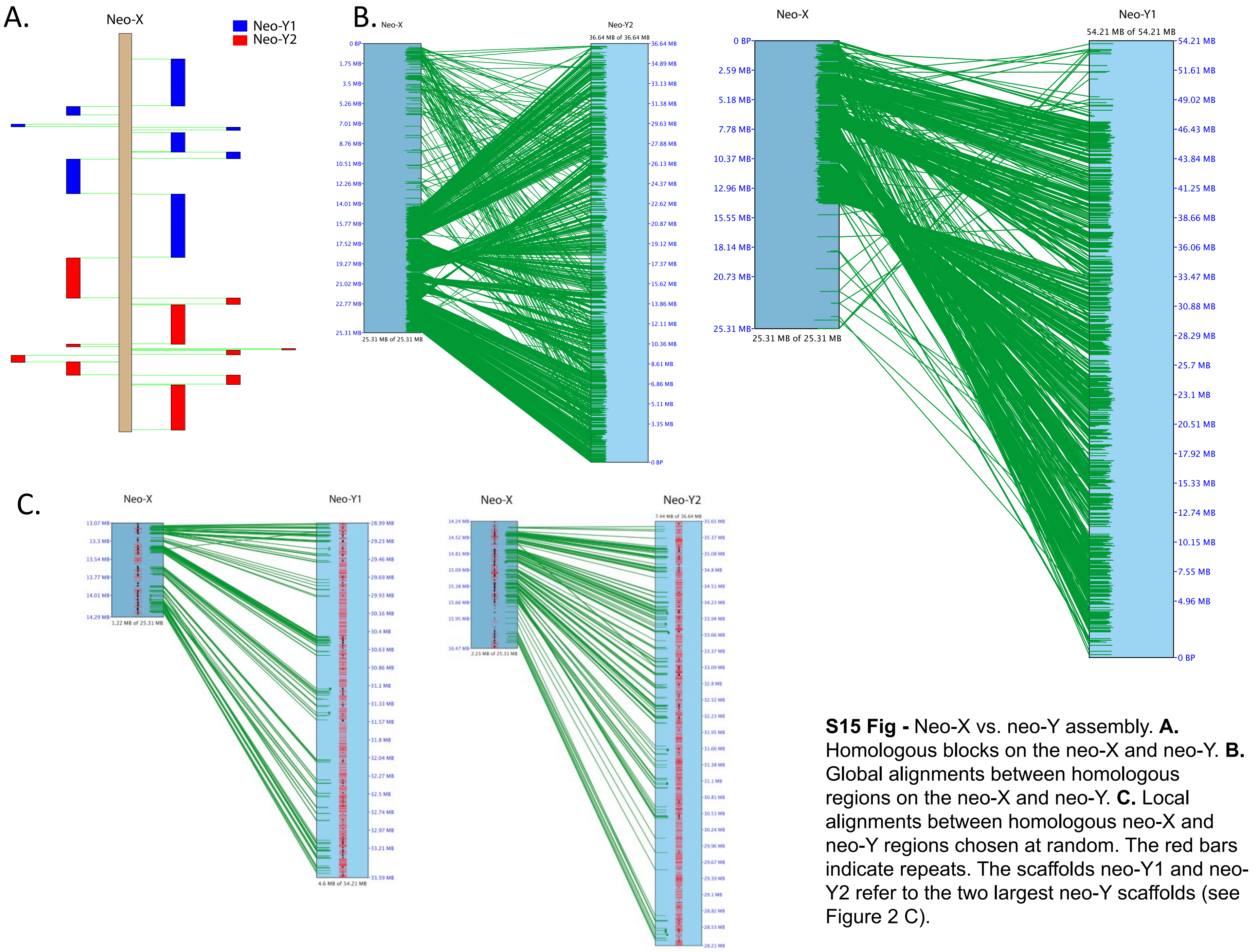

S15 Fig - Neo-X vs. neo-Y assembly. **A.** Homologous blocks on the neo-X and neo-Y. **B.** Global alignments between homologous regions on the neo-X and neo-Y. **C.** Local alignments between homologous neo-X and neo-Y regions chosen at random. The red bars indicate repeats. The scaffolds neo-Y1 and neo-Y2 refer to the two largest neo-Y scaffolds (see Figure 2 C).
