# Supplementary material for: De novo assembly of a young Drosophila Y chromosome using single-molecule sequencing and chromatin conformation capture
Source: PLoS Biol. 2018 Jul 30;16(7):e2006348. doi: 10.1371/journal.pbio.2006348 (PMC6117089; doi:10.1371/journal.pbio.2006348)
Supplement: S16 Fig — The pie chart insert shows the relative types of different repeats across chromosomes, and the bar charts show the absolute number of bases masked for the various repeats across chromosomes. Underlying data can be found in S1 Data. (PDF) [file pbio.2006348.s016.pdf]

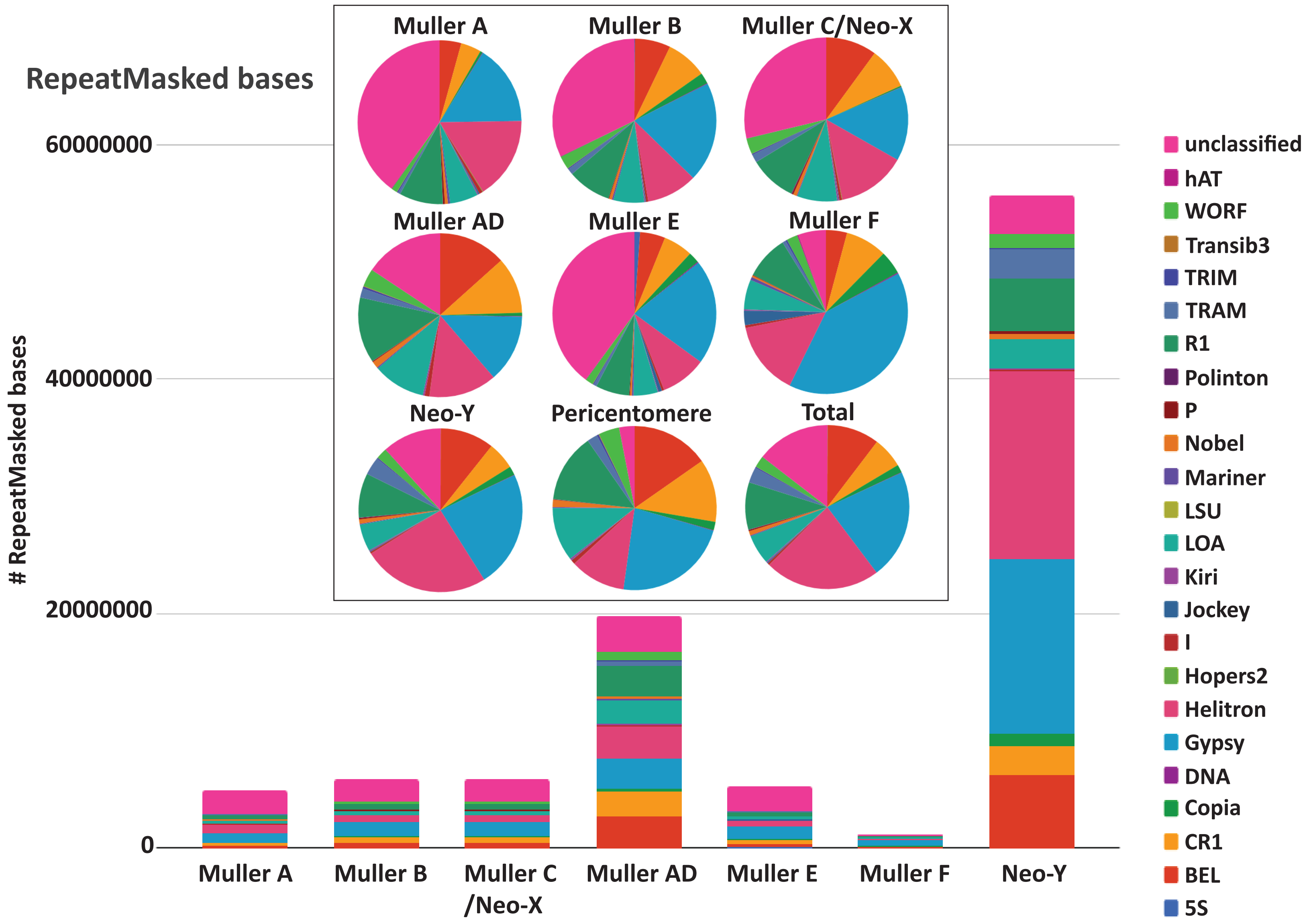

S16 Fig – Distribution of different repeat types across the *D. miranda* genome. The pie chart insert shows the relative types of different repeats across chromosomes, and the bar charts show the absolute number of bases masked for the various repeats across chromosomes. Underlying data can be found in S1_Data.xlsx.
