# Supplementary material for: De novo assembly of a young Drosophila Y chromosome using single-molecule sequencing and chromatin conformation capture
Source: PLoS Biol. 2018 Jul 30;16(7):e2006348. doi: 10.1371/journal.pbio.2006348 (PMC6117089; doi:10.1371/journal.pbio.2006348)
Supplement: S17 Fig — A. Comparison between D. pseudoobscura and D. miranda dot chromosome reveals an inversion between species involving the translocated ancestral Y region. B. BAC clone S513-N704 spans the Y-dot translocation and contains both ancestral Y genes (kl-3) as well as genes from Muller element F (shown in orange). C. H3K9me3 enrichment at genes on the dot that are derived from the ancestral Y versus Muller element F. D. Ancestral Y genes show testis-specific expression. Underlying data can be found in S1 Data. BAC, bacterial artificial chromosome; H3K9me3, trimethylation of histone 3 lysine 9. (PDF) [file pbio.2006348.s017.pdf]

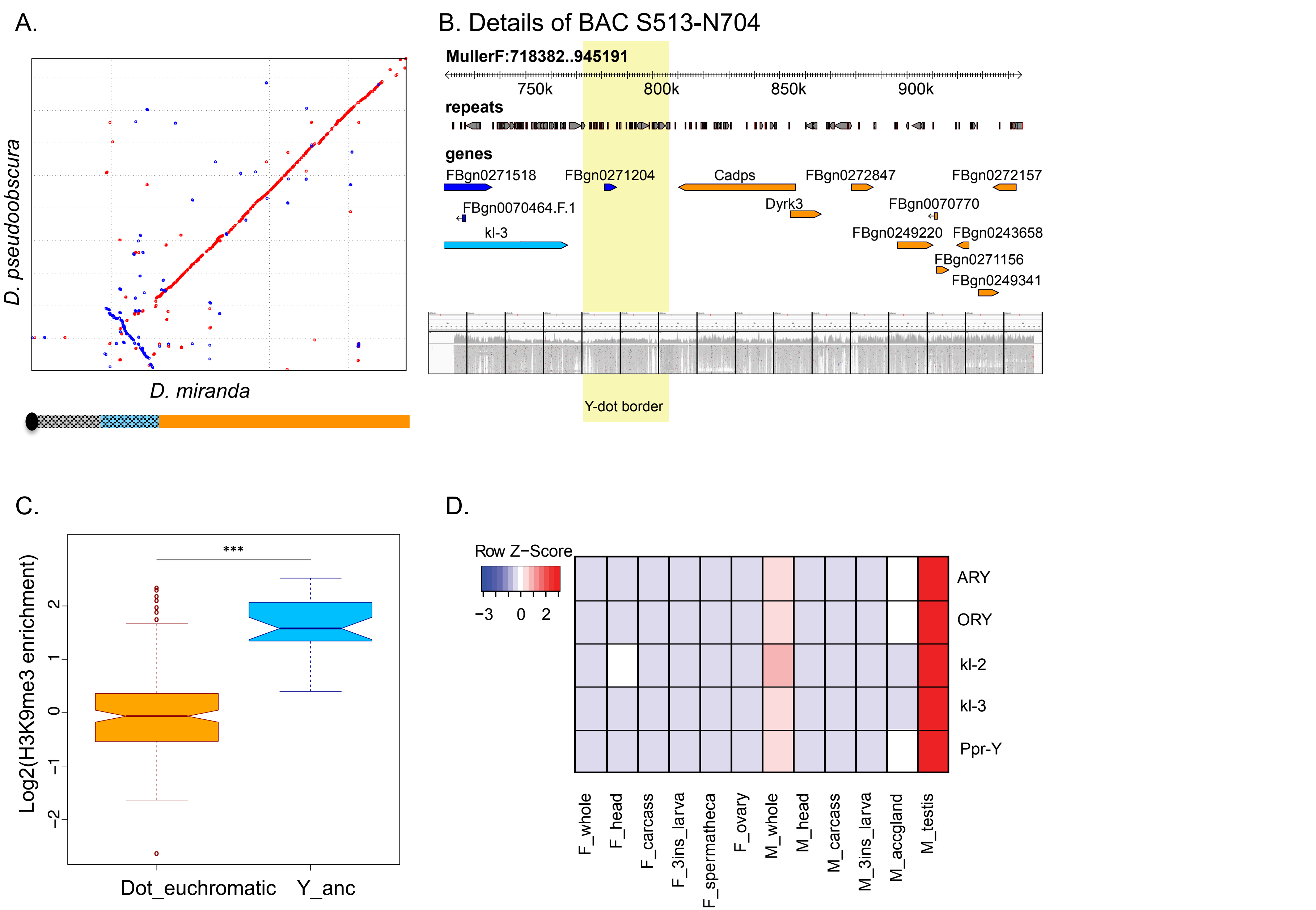


**S17 Fig** – Single-copy Y genes have been translocated onto the dot chromosome. **A.** Comparison between *D. pseudoobscura* and *D. miranda* dot chromosome reveals an inversion between species involving the translocated ancestral Y region. **B.** BAC clone S513-N704 spans the Y-dot translocation and contains both ancestral Y genes (*kl-3*) as well as genes from Muller element F (shown in orange). **C.** H3K9me3 enrichment at genes on the dot that are derived from the ancestral Y vs. Muller element F. **D.** Ancestral Y genes show testis-specific expression. Underlying data can be found in S1_Data.xlsx.
