# Supplementary material for: De novo assembly of a young Drosophila Y chromosome using single-molecule sequencing and chromatin conformation capture
Source: PLoS Biol. 2018 Jul 30;16(7):e2006348. doi: 10.1371/journal.pbio.2006348 (PMC6117089; doi:10.1371/journal.pbio.2006348)
Supplement: S18 Fig — A total of 2,407,465 reads with an average length of 12,818 bp and NR50 of 17,116 bp were collected. NR50, read length such that 50% of the total sequence is contained within reads of this length or longer. (PDF) [file pbio.2006348.s018.pdf]

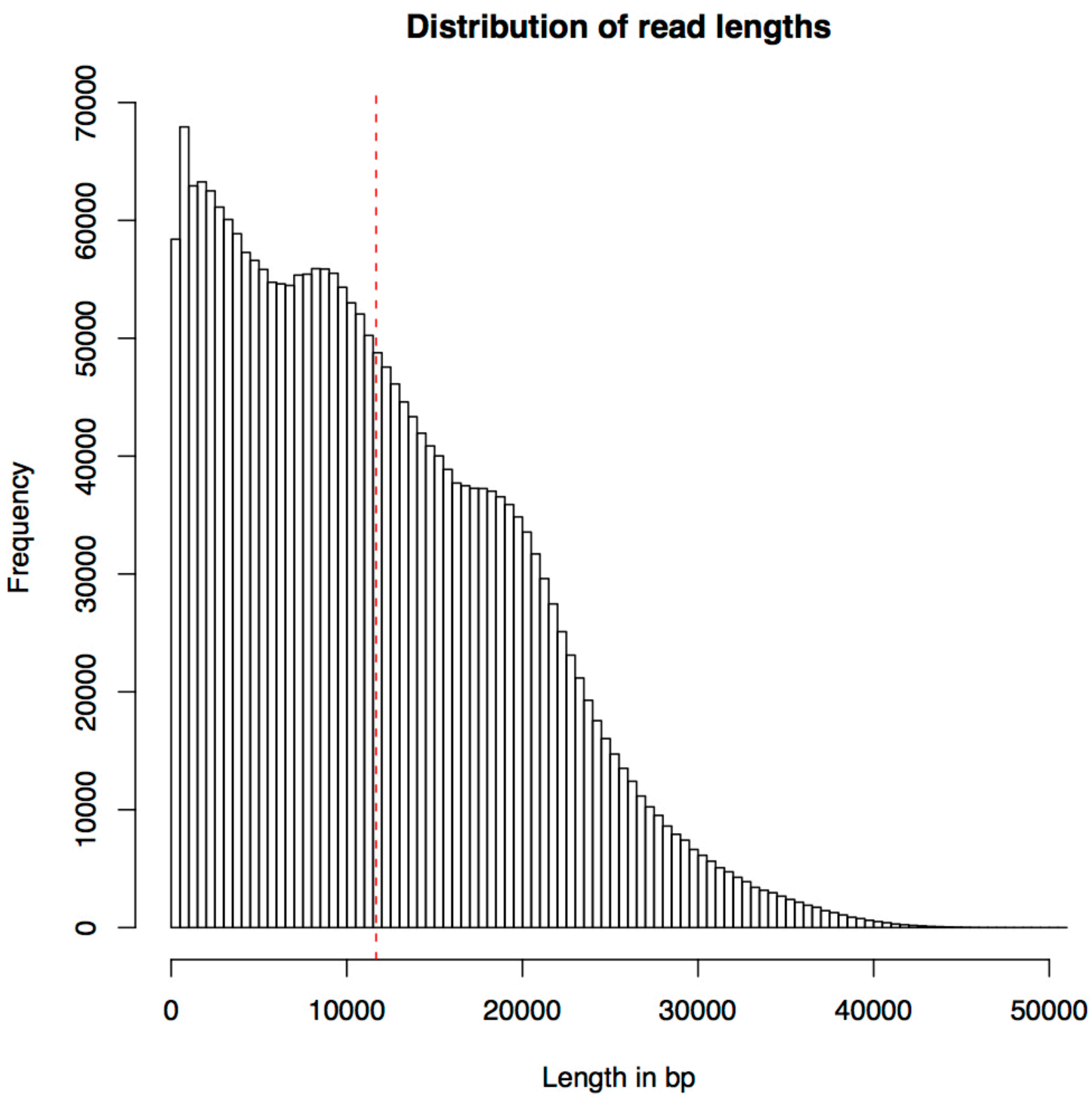

S18 Fig – Length distribution of PacBio reads. A total of 2,407,465 reads with an average length of 12,818-bp and NR50 of 17,116-bp were collected.
