# Supplementary material for: De novo assembly of a young Drosophila Y chromosome using single-molecule sequencing and chromatin conformation capture
Source: PLoS Biol. 2018 Jul 30;16(7):e2006348. doi: 10.1371/journal.pbio.2006348 (PMC6117089; doi:10.1371/journal.pbio.2006348)
Supplement: S19 Fig — A. Molecule length distribution, B. molecule length versus molecule average intensity. (PDF) [file pbio.2006348.s019.pdf]

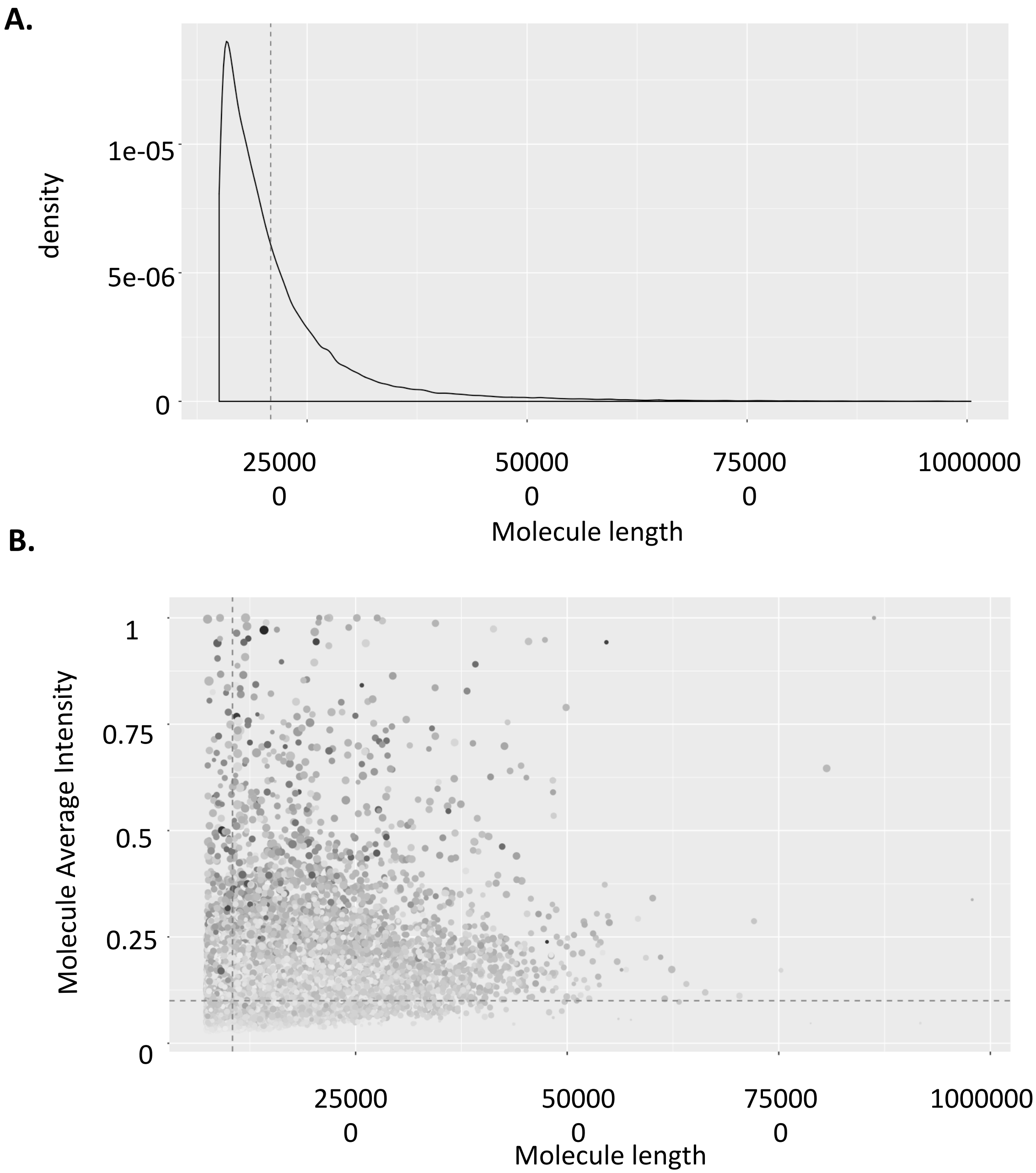

S19 Fig – BioNano data. **A.** Molecule length distribution **B.** Molecule Length vs. Molecule Average Intensity.
