# Supplementary material for: De novo assembly of a young Drosophila Y chromosome using single-molecule sequencing and chromatin conformation capture
Source: PLoS Biol. 2018 Jul 30;16(7):e2006348. doi: 10.1371/journal.pbio.2006348 (PMC6117089; doi:10.1371/journal.pbio.2006348)
Supplement: S1 Table — (PDF) [file pbio.2006348.s020.pdf]

S1 Table. Data used for the current assembly

Sequencing	sample	Number of reads	Total amount of data (No. of bases)
Illumina HiSeq	MSH22 female	32801952 pairs	6.6Gb
Illumina HiSeq	MSH22 female	8244007 pairs	1.6Gb
Illumina HiSeq	MSH22 female	11461680 pairs	2.3Gb
Illumina HiSeq	MSH22 male	12494994 pairs	2.5Gb
Illumina HiSeq	MSH22 male	9724346 pairs	1.9Gb
Pacbio	MSH22 male	2407465 reads	28Gb
Bionano	MSH22 male	90977 Molecules	19.9Gb
BAC clones	MSH22 male	365505123 pairs	73.1Gb
Hi-C	MSH22 male	27990598 pairs	5.6Gb
Hi-C	MSH22 female	38433349 pairs	7.7Gb
