# Supplementary material for: De novo assembly of a young Drosophila Y chromosome using single-molecule sequencing and chromatin conformation capture
Source: PLoS Biol. 2018 Jul 30;16(7):e2006348. doi: 10.1371/journal.pbio.2006348 (PMC6117089; doi:10.1371/journal.pbio.2006348)
Supplement: S2 Table — (PDF) [file pbio.2006348.s021.pdf]

S2 Table. Manual corrections of assembly

A. Parts of contigs removed due to false duplications introduced by misassembly

Contig ID	Start posn.	End posn.
mpm220	2040000	End
mpm7	255400	End
mpm256	0	230000
mpm170	0	520000
mpm159	1100000	End
mpm172	0	120000
mpm11	0	140000
mpm184	0	420000
mpm4	0	220000
mpm227	330000	End
mpm259	540000	End
mpm20	0	160000
mpm241	45000	120000

B. Contigs split based on the HIC Maps

Contig ID	Start	End	Comment
mpm191	0	3000000	Broke into two
	3000000	End	
mpm228	0	550000	Broke into three
	550000	975000	
	975000	End	
mpm164	0	800000	Broke into two
	800000	End	
mpm181	0	575000	Broke into two
	575000	End	
mpm166	0	550000	Broke into two
	550000	End	
mpm169	0	2475000	Broke into two
	2475000	End	
mpm237	0	2050000	Broke into two
	2050000	End	
