# Supplementary material for: De novo assembly of a young Drosophila Y chromosome using single-molecule sequencing and chromatin conformation capture
Source: PLoS Biol. 2018 Jul 30;16(7):e2006348. doi: 10.1371/journal.pbio.2006348 (PMC6117089; doi:10.1371/journal.pbio.2006348)
Supplement: S3 Table — Note that the published D. miranda genome is substantially smaller and lacks an assembly of repeat-rich regions and the Y/neo-Y chromosome. (PDF) [file pbio.2006348.s022.pdf]

S3 Table. Structural variants identified using Lumpyexpress, by mapping two male MSH22 Illumina libraries back to the reference genomes (626bp and 915bp insert sizes). Note that the published *D. miranda* genome is substantial ly smaller, and lacks an assembly of repeat-rich regions and the Y/neo-Y chromosome.

Assembly	BND	DEL	DUP	INV	Errors per Mb
Canu	108	206	10	2	1.1
Polished Falcon	224	229	35	10	1.82
Quickmerged	64	348	17	2	1.46
Pacbio + HiC	62	292	17	2	1.29
Old *D. miranda* assembly (Chromosomes + unplaced scaffolds)	14248	5780	1430	77	154.16
Old *D.miranda* assembly (Chromosomes only)	17764	6075	1703	117	187.66
D.miranda current	36	229	8	4	0.96

*BND = trans-contig association, DEL = deletion, DUP = duplication, INV = inversion
