# Supplementary material for: De novo assembly of a young Drosophila Y chromosome using single-molecule sequencing and chromatin conformation capture
Source: PLoS Biol. 2018 Jul 30;16(7):e2006348. doi: 10.1371/journal.pbio.2006348 (PMC6117089; doi:10.1371/journal.pbio.2006348)
Supplement: S4 Table — NGS, next-generation sequencing. (PDF) [file pbio.2006348.s023.pdf]

S4 Table. BioNano Data: Hybrid scaffold coverage by BioNano contigs and our next-generation sequencing (NGS) contigs

Mapping our NGS contigs to the Hybridscaffold contigs

refContig	refContig_size	query_cov_bp	query_unique_cov_bp	query_unique_cov_percent
74	658745	652690	652690	99.08
(Muller C) 11	25,306,201	25286725	25286725	99.92
(NeoY2) 75	36,637,378	36605472	36605472	99.91
7	164344.7	160360.5	160360.5	97.58
(Muller F) 9	2366016	2281611	2281611	96.43
(Muller E) 15	35263102	35199171.5	35199171.5	99.82
14	504350	440865	440865	87.41
(YD) 103	10691914	10675716	10675716	99.85
77	1459333	1403370	1403370	96.17
(Muller AD) 46	52406784	52401703	52401703	99.99
(NeoY1) 76	54206685	54187229	54187229	99.96
(Muller B) 10	32539883	32260759	32260759	99.14
(Muller A) 16	25342444	25341081	25341081	99.99

Mapping BioNano contigs to the Hybridscaffold contigs

refContig	refContig_size	query_cov_bp	query_unique_cov_bp	query_unique_cov_percent
74	658745	366445	366445	55.63
(Muller C) 11	25306201	15681881.5	15451734.5	61.06
(NeoY2) 75	36637378	12332602	12195516	33.29
7	164344.7	160360.5	160360.5	97.58
(Muller F) 9	2366016	1810021.5	1723909	72.86
(Muller E) 15	35263102	30059227	29526484	83.73
14	504350	88972	88972	17.64
(YD) 103	10691914	0	0	0
77	1459333	272901	272901	18.7
(Muller AD) 46	52406784	18870397	18673578	35.63
(NeoY1)76	54206685	11541163	11441729	21.11
(Muller B) 10	32539883	28697745.5	27852321	85.59
(Muller A) 16	25342444	16468657	16345226.5	64.5
