# Supplementary material for: De novo assembly of a young Drosophila Y chromosome using single-molecule sequencing and chromatin conformation capture
Source: PLoS Biol. 2018 Jul 30;16(7):e2006348. doi: 10.1371/journal.pbio.2006348 (PMC6117089; doi:10.1371/journal.pbio.2006348)
Supplement: S5 Table — BAC, bacterial artificial chromosome. (PDF) [file pbio.2006348.s024.pdf]

S5 Table. Summary of mapping location of BAC clone data

chromosome	# BAC's mapping
Muller A	20
Muller AD	51
Muller B	80
Muller E	84
neo-X	28
Y/neo-Y	92
unknown	6

*11 BAC clones map to 2 or 3 locations
