# Supplementary material for: De novo assembly of a young Drosophila Y chromosome using single-molecule sequencing and chromatin conformation capture
Source: PLoS Biol. 2018 Jul 30;16(7):e2006348. doi: 10.1371/journal.pbio.2006348 (PMC6117089; doi:10.1371/journal.pbio.2006348)
Supplement: S6 Table — BAC, bacterial artificial chromosome. (PDF) [file pbio.2006348.s025.pdf]

S6 Table. Mapping location of BAC clone data

BAC barcode	location	start	end	total_bp	% repeat
S513_N704	unmapped	718382	945190	226808	40
S505_N721	unmapped	1672181	1848995	176814	20
S502_N705	unmapped	1883984	2088170	204186	19
S514_N711	unmapped	370373	543426	173053	46
S505_N704	unmapped	118241	348335	230094	87
S501_N715	unmapped	299643	487609	217276	88
S508_N704	Muller A	5478663	5721440	242777	92
S504_N719	Muller A	6330324	6519789	189465	11
S509_N714	Muller A	10537187	10738539	201352	4
S510_N717	Muller A	10689564	10878963	189399	15
S503_N719	Muller A	10978898	11207533	228635	5
S507_N719	Muller A	11059379	11344851	285472	15
S511_N715	Muller A	13609670	13807745	198075	2
S516_N721	Muller A	14610608	14798383	187775	5
S504_N708	Muller A	15818863	16004970	186107	10
S510_N706	Muller A	16543024	16741012	197988	3
S506_N704	Muller A	16747468	16964023	216555	4
S510_N712	Muller A	16966765	17115926	149161	4
S515_N715	Muller A	17067240	17263532	196292	7
S515_N702	Muller A	17208487	17410884	202397	4
S501_N718	Muller A	17692299	17915872	223573	11
S511_N707	Muller A	18238486	18427161	188675	5
S508_N721	Muller A	21001285	21196642	195357	4
S513_N717	Muller A	23602555	23797024	194469	6
S514_N708	Muller A	4245098	4509718	264620	68
S505_N714	Muller A	3329276	3478646	149370	35
S516_N707	Muller AD	161921	337849	175928	6
S515_N724	Muller AD	418372	626589	208217	5
S508_N708	Muller AD	792490	1000722	208232	4
S513_N719	Muller AD	2879144	3085474	206330	14
S509_N705	Muller AD	3023332	3262316	238984	12
S512_N706	Muller AD	4077631	4285615	207984	5
S503_N711	Muller AD	4658992	4851682	192690	3
S513_N722	Muller AD	6553936	6736943	183007	4
S515_N721	Muller AD	7332648	7533273	200625	8
S509_N702	Muller AD	9598804	9816006	217202	2
S508_N702	Muller AD	10490149	10676494	186345	4
S515_N711	Muller AD	10747991	10934244	186253	5
S512_N720	Muller AD	11344878	11538084	193206	1

S504_N718	Muller AD	13912219	14098607	186388	3
S507_N704	Muller AD	15161833	15356457	194624	5
S514_N710	Muller AD	15550808	15757650	206842	6
S511_N704	Muller AD	16974601	17164660	190059	4
S515_N718	Muller AD	17558039	17743114	185075	4
S504_N720	Muller AD	20099035	20328484	229449	3
S513_N713	Muller AD	20970478	21159681	189203	8
S512_N721	Muller AD	21418116	21644736	226620	3
S514_N715	Muller AD	22224659	22412829	188170	7
S504_N710	Muller AD	23650667	23857932	207265	2
S508_N724	Muller AD	24062359	24280656	218297	2
S516_N719	Muller AD	24596644	24784485	187841	6
S507_N701	Muller AD	24782684	24971500	188816	5
S515_N719	Muller AD	26292506	26508102	215596	34
S504_N721	Muller AD	26317975	26519962	201987	38
S514_N706	Muller AD	27727132	27960501	233369	76
S510_N716	Muller AD	30493872	30694756	200884	20
S504_N703	Muller AD	31968296	32173736	205440	51
S510_N721	Muller AD	32392076	32581367	189291	72
S506_N711	Muller AD	32542801	32760739	217938	70
S501_N719	Muller AD	33204964	33421877	216913	85
S503_N707	Muller AD	33437931	33644750	206819	79
S516_N703	Muller AD	34415006	34626075	211069	91
S516_N715	Muller AD	34485564	34571705	86141	92
S509_N717	Muller AD	34604826	34795341	190515	76
S513_N707	Muller AD	34889150	35094463	205313	78
S511_N722	Muller AD	42169748	42377439	207691	85
S505_N708	Muller AD	42896378	43109794	213416	92
S512_N708	Muller AD	43925431	44150324	224893	84
S512_N711	Muller AD	44143147	44424872	281725	79
S515_N706	Muller AD	44871629	45069585	197956	86
S505_N717	Muller AD	45478930	45663912	184982	74
S511_N717	Muller AD	50449743	50658016	208273	82
S514_N723	Muller AD	51387998	51616547	228549	85
S506_N701	Muller AD	51423804	51632782	208978	85
S507_N718	Muller AD	51545244	51777844	232600	86
S507_N717	Muller AD	26317975	26519962	201987	38
S515_N708	Muller AD	42208598	42377762	169164	84
S501_N712	Muller B	645950	900967	255017	99
S512_N714	Muller B	1129205	1330854	201649	80
S507_N708	Muller B	1753858	1962607	208749	85
S503_N715	Muller B	1940845	2155184	214339	84
S505_N723	Muller B	2413264	2596360	183096	75

S508_N720	Muller B	2505758	2746763	241005	80
S507_N720	Muller B	3115362	3333717	218355	69
S510_N715	Muller B	3139129	3358522	219393	68
S510_N714	Muller B	3139183	3358424	219241	68
S506_N703	Muller B	3354274	3547330	193056	39
S502_N711	Muller B	3369698	3563168	193470	39
S505_N710	Muller B	5307192	5493004	185812	2
S513_N705	Muller B	5426552	5638689	212137	4
S513_N706	Muller B	5852170	6036199	184029	3
S515_N709	Muller B	6022800	6207874	185074	5
S502_N710	Muller B	6899548	7106136	206588	3
S506_N715	Muller B	9028925	9236282	207357	8
S512_N719	Muller B	9310992	9488246	177254	5
S510_N701	Muller B	10591302	10777241	185939	6
S512_N723	Muller B	10706272	10896938	190666	2
S507_N705	Muller B	11061138	11259863	198725	2
S511_N714	Muller B	11259061	11464075	205014	4
S503_N714	Muller B	11349270	11568513	219243	4
S501_N703	Muller B	11408896	11607845	198949	5
S506_N719	Muller B	11509331	11747438	238107	22
S506_N706	Muller B	11697745	11921938	224193	27
S509_N722	Muller B	11802582	12022493	219911	7
S502_N702	Muller B	13228231	13414384	186153	10
S504_N724	Muller B	13407955	13605992	198037	5
S507_N702	Muller B	13726945	13952690	225745	2
S511_N710	Muller B	13813678	14019269	205591	4
S509_N713	Muller B	14707576	14910911	203335	4
S511_N724	Muller B	14708524	14918594	210070	4
S508_N703	Muller B	14852870	15048107	195237	3
S513_N703	Muller B	14995687	15195284	199597	4
S510_N707	Muller B	16114731	16318958	204227	3
S512_N709	Muller B	16151473	16348986	197513	3
S501_N721	Muller B	17407757	17603226	195469	4
S501_N711	Muller B	17782695	17981092	198397	2
S510_N702	Muller B	18023489	18245152	221663	7
S503_N704	Muller B	18559870	18764046	204176	10
S502_N724	Muller B	18666187	18856056	189869	13
S508_N713	Muller B	18919430	19125449	206019	18
S504_N706	Muller B	19794662	19991051	196389	7
S513_N718	Muller B	19877992	20086644	208652	6
S503_N702	Muller B	20043678	20245290	201612	3
S502_N721	Muller B	20127455	20314531	187076	3
S506_N722	Muller B	20127455	20314531	187076	3

S508_N716	Muller B	20707986	20919970	211984	3
S502_N708	Muller B	20922696	21114694	191998	8
S509_N715	Muller B	21233359	21430597	197238	4
S506_N720	Muller B	21859204	22043011	183807	6
S509_N712	Muller B	22014099	22229141	215042	3
S501_N714	Muller B	22097678	22300199	202521	2
S505_N703	Muller B	23558752	23746252	187500	1
S505_N706	Muller B	23570716	23757074	186358	1
S504_N712	Muller B	23802019	23995031	193012	7
S508_N718	Muller B	24144126	24333337	189211	6
S512_N710	Muller B	25111230	25302143	190913	8
S503_N701	Muller B	25879178	26092684	213506	10
S504_N705	Muller B	26547242	26748321	201079	5
S501_N702	Muller B	26547243	26732960	185717	5
S515_N703	Muller B	26795278	26982519	187241	3
S501_N716	Muller B	27036999	27246489	209490	1
S502_N713	Muller B	27438363	27622963	184600	3
S503_N708	Muller B	27956618	28153514	196896	6
S516_N701	Muller B	28208048	28402110	194062	6
S516_N713	Muller B	28208079	28402104	194025	6
S501_N706	Muller B	28936772	29153186	216414	5
S516_N711	Muller B	29493256	29692814	199558	6
S514_N702	Muller B	29995675	30181148	185473	2
S514_N707	Muller B	30178213	30369630	191417	7
S501_N717	Muller B	30720714	30918373	197659	10
S512_N715	Muller B	31116625	31305064	188439	0
S509_N703	Muller B	31305063	31519063	214000	3
S504_N715	Muller B	32092927	32290306	197379	1
S503_N720	Muller B	32185201	32381318	196117	2
S503_N718	Muller B	16069687	16279767	210080	3
S508_N706	Muller B	13022659	13062372	39713	7
S510_N719	Muller B	16807886	16907765	99879	3
S514_N716	Muller E	459945	663190	203245	6
S502_N722	Muller E	1162898	1365518	202620	1
S510_N709	Muller E	1601146	1800185	199039	3
S504_N723	Muller E	2249879	2459734	209855	6
S501_N701	Muller E	3232617	3417324	184707	2
S513_N701	Muller E	3784231	3988423	204192	3
S512_N705	Muller E	4075059	4275636	200577	1
S507_N716	Muller E	4314592	4510971	196379	4
S513_N712	Muller E	4344175	4568296	224121	2
S503_N717	Muller E	4450164	4641552	191388	2
S515_N710	Muller E	4876334	5079420	203086	3

S513_N711	Muller E	4916088	5149286	233198	6
S507_N715	Muller E	5378861	5619256	240395	4
S514_N712	Muller E	5653977	5860355	206378	6
S506_N710	Muller E	5946427	6147634	201207	3
S507_N712	Muller E	6906892	7112858	205966	4
S509_N709	Muller E	7361520	7559785	198265	6
S505_N707	Muller E	8150096	8339894	189798	2
S503_N706	Muller E	9244985	9428407	183422	1
S504_N716	Muller E	10432534	10644210	211676	2
S511_N716	Muller E	10477134	10663483	186349	1
S508_N710	Muller E	10649207	10869014	219807	7
S507_N724	Muller E	11354458	11555920	201462	3
S506_N708	Muller E	11507094	11706095	199001	3
S513_N709	Muller E	11864223	12069043	204820	3
S513_N708	Muller E	12207300	12422395	215095	4
S514_N703	Muller E	13272817	13461532	188715	3
S510_N720	Muller E	14073562	14305155	231593	11
S510_N708	Muller E	14430560	14625129	194569	4
S507_N714	Muller E	14631792	14815849	184057	8
S511_N705	Muller E	14884651	15099668	215017	8
S506_N709	Muller E	15830329	16015172	184843	2
S501_N708	Muller E	17925403	18053108	127705	24
S508_N723	Muller E	18220211	18423581	203370	2
S504_N717	Muller E	18897986	19097995	200009	18
S514_N721	Muller E	19358843	19569954	211111	43
S501_N710	Muller E	19925979	20126318	200339	43
S512_N703	Muller E	20394796	20578236	183440	3
S505_N702	Muller E	20650357	20844751	194394	12
S506_N717	Muller E	21207693	21375753	168060	4
S515_N720	Muller E	21605197	21779804	174607	3
S506_N702	Muller E	22418405	22636139	217734	2
S514_N709	Muller E	22943442	23141115	197673	3
S504_N702	Muller E	23040459	23231930	191471	2
S513_N710	Muller E	23345770	23587203	241433	5
S516_N720	Muller E	23681496	23873409	191913	6
S515_N701	Muller E	25307819	25516951	209132	2
S512_N716	Muller E	25855392	26049744	194352	8
S511_N708	Muller E	26002791	26200087	197296	19
S512_N712	Muller E	26200044	26404740	204696	9
S509_N721	Muller E	26233885	26412961	179076	10
S509_N708	Muller E	26363739	26579013	215274	10
S503_N712	Muller E	26386866	26603295	216429	8
S515_N716	Muller E	26606889	26807106	200217	6

S503_N724	Muller E	26650803	26807162	156359	6
S514_N719	Muller E	26793718	26990118	196400	12
S509_N706	Muller E	27035759	27243550	207791	8
S507_N703	Muller E	27713846	27909095	195249	5
S515_N723	Muller E	27739744	27944450	204706	4
S503_N713	Muller E	28159283	28352817	193534	3
S516_N705	Muller E	28748769	28972959	224190	1
S502_N704	Muller E	29003074	29203004	199930	7
S511_N702	Muller E	29303386	29519200	215814	4
S502_N720	Muller E	29396310	29581451	185141	5
S501_N713	Muller E	29455567	29649363	193796	6
S514_N720	Muller E	29494494	29696124	201630	6
S514_N701	Muller E	29784428	29992247	207819	1
S511_N701	Muller E	30653037	30843678	190641	6
S508_N717	Muller E	31006799	31194710	187911	5
S507_N723	Muller E	31006799	31194715	187916	5
S503_N710	Muller E	31081726	31265909	184183	6
S510_N705	Muller E	31293521	31499493	205972	6
S511_N712	Muller E	31638569	31849373	210804	5
S502_N706	Muller E	31660786	31855042	194256	7
S501_N709	Muller E	31842642	32044025	201383	11
S512_N722	Muller E	32148347	32330435	182088	3
S504_N707	Muller E	32166529	32358754	192225	3
S504_N709	Muller E	32983438	33205797	222359	3
S513_N724	Muller E	33396016	33626588	230572	92
S502_N715	Muller E	33430990	33642165	211175	91
S502_N712	Muller E	34269546	34481860	212314	98
S512_N704	Muller E	34533834	34729690	195856	99
S506_N718	Muller E	34537800	34726298	188498	99
S508_N719	Muller E	18220211	18423581	203370	2
S509_N724	neo-X	301560	513679	212119	90
S505_N715	neo-X	436229	652316	216087	83
S512_N724	neo-X	1150320	1367177	216857	77
S509_N710	neo-X	1560384	1747721	187337	65
S515_N707	neo-X	2937165	3081798	144633	91
S507_N711	neo-X	3002090	3215569	213479	76
S501_N724	neo-X	4904258	5115555	211297	24
S505_N712	neo-X	5935473	6128602	193129	27
S511_N721	neo-X	8376821	8582793	205972	4
S508_N712	neo-X	10759209	10957839	198630	23
S505_N713	neo-X	11427828	11635003	207175	15
S515_N713	neo-X	12597863	12821890	224027	15
S508_N709	neo-X	12997801	13185900	188099	5

S509_N711	neo-X	14851101	15040849	189748	3
S503_N703	neo-X	14971249	15179394	208145	10
S509_N720	neo-X	15617056	15821521	204465	2
S502_N709	neo-X	15954579	16166838	212259	0
S504_N704	neo-X	16226716	16452168	225452	4
S511_N703	neo-X	18042492	18227141	184649	3
S506_N707	neo-X	18907961	19096774	188813	4
S502_N718	neo-X	21176431	21358295	181864	2
S505_N720	neo-X	21665617	21848542	182925	5
S503_N722	neo-X	22187248	22294684	107436	0
S503_N705	neo-X	22663113	22857194	194081	2
S513_N716	neo-X	23426280	23604359	178079	2
S504_N711	neo-X	23700592	23908955	208363	3
S514_N717	neo-X	24501863	24703216	201353	4
S510_N703	neo-X	24985934	25188106	202172	2
S502_N714	neo-Y	1803144	2070251	267107	69
S514_N713	neo-Y	2709711	2911103	201392	77
S513_N714	neo-Y	3039803	3113254	73451	49
S504_N722	neo-Y	3229669	3451121	221452	69
S515_N705	neo-Y	3655552	3838040	182488	61
S501_N705	neo-Y	3838034	4016860	178826	72
S503_N721	neo-Y	3904746	4119023	214277	78
S516_N704	neo-Y	5084225	5272705	188480	69
S516_N716	neo-Y	5084282	5311227	226945	72
S511_N713	neo-Y	5312422	5530588	218166	49
S507_N713	neo-Y	5322196	5537713	215517	48
S516_N710	neo-Y	6111973	6322935	210962	63
S505_N711	neo-Y	6134273	6329145	194872	78
S505_N719	neo-Y	7113161	7285835	172674	55
S516_N708	neo-Y	7446634	7683459	236825	63
S510_N704	neo-Y	8040027	8229902	189875	76
S515_N717	neo-Y	9253057	9479208	226151	67
S516_N722	neo-Y	10006932	10216328	209396	66
S505_N724	neo-Y	10844824	11049846	205022	63
S505_N722	neo-Y	10845123	11049841	204718	63
S513_N720	neo-Y	10974618	11199649	225031	59
S502_N723	neo-Y	11301916	11520327	218411	69
S514_N704	neo-Y	11747776	11964188	216412	55
S505_N701	neo-Y	12010485	12228271	217786	67
S501_N720	neo-Y	13098245	13178555	80310	69
S516_N706	neo-Y	13251596	13488186	236590	81
S516_N718	neo-Y	13251603	13488186	236583	81
S506_N705	neo-Y	13515504	13641563	126059	89

S506_N716	neo-Y	14055651	14274883	219232	86
S515_N722	neo-Y	14148264	14407734	259470	75
S506_N713	neo-Y	16282941	16459962	177021	73
S508_N705	neo-Y	17127650	17343265	215615	66
S515_N704	neo-Y	18129386	18302657	173271	71
S514_N724	neo-Y	18405730	18607160	201430	70
S502_N716	neo-Y	19316828	19533457	216629	56
S508_N714	neo-Y	21233862	21425349	191487	69
S512_N707	neo-Y	24738817	24957462	218645	87
S510_N722	neo-Y	26750398	26962573	212175	61
S509_N719	neo-Y	27777862	27947682	169820	68
S513_N715	neo-Y	27830258	28061990	231732	71
S506_N721	neo-Y	27941261	28133534	192273	71
S509_N718	neo-Y	28024246	28222580	198334	66
S505_N709	neo-Y	28060764	28308647	247883	55
S503_N716	neo-Y	28411620	28597526	185906	58
S511_N720	neo-Y	29229862	29518585	288723	58
S515_N712	neo-Y	29511621	29750227	238606	61
S513_N702	neo-Y	29729434	29926625	197191	74
S508_N711	neo-Y	30027122	30241041	213919	92
S502_N701	neo-Y	30805453	31038872	233419	71
S502_N703	neo-Y	31353545	31488050	134505	84
S512_N702	neo-Y	32073086	32298497	225411	74
S511_N711	neo-Y	32220024	32418587	198563	74
S509_N701	neo-Y	32305731	32523158	217427	71
S508_N701	neo-Y	34193218	34393870	200652	85
S501_N707	neo-Y	35572215	35802148	229933	73
S501_N722	neo-Y	38710134	38918467	208333	76
S507_N721	neo-Y	38832970	39037056	204086	72
S504_N713	neo-Y	39159539	39362673	203134	78
S504_N714	neo-Y	39575288	39788781	213493	70
S502_N707	neo-Y	40092611	40290570	197959	62
S501_N723	neo-Y	40362297	40548597	186300	54
S511_N723	neo-Y	41492723	41717228	224505	72
S511_N706	neo-Y	41520700	41755134	234434	72
S507_N709	neo-Y	41618205	41817522	199317	80
S511_N719	neo-Y	41796388	42008025	211637	72
S516_N723	neo-Y	42836503	42964724	128221	54
S513_N723	neo-Y	43718115	43917597	199482	76
S512_N718	neo-Y	44234005	44436255	202250	52
S508_N722	neo-Y	45150288	45300761	150473	93
S503_N709	neo-Y	45582023	45788561	206538	92
S516_N702	neo-Y	46239532	46438708	199176	85

S516_N714	neo-Y	46239532	46438708	199176	85
S506_N714	neo-Y	46355974	46401123	45149	91
S515_N714	neo-Y	46553454	46757519	204065	83
S510_N710	neo-Y	48924942	49158759	233817	93
S508_N707	neo-Y	49448608	49637061	188453	88
S508_N715	neo-Y	49748052	49947839	199787	90
S506_N724	neo-Y	51684200	51887527	203327	91
S514_N705	neo-Y	52081191	52335932	254741	85
S502_N717	neo-Y	19467336	19667372	200036	56
S510_N713	neo-Y	5512025	5566452	54427	84
S510_N718	neo-Y	13709055	13830105	121050	98
S513_N721	neo-Y	7678171	7844115	165944	55
S514_N714	neo-Y	51863727	51987674	123947	95
S516_N709	neo-Y	502587	668687	166100	82
S512_N701	neo-Y	20314165	20444797	130632	93
S507_N710	Y or neo-Y	194882	443439	248557	80
S512_N717	Y or neo-Y	200558	410343	209785	81
S512_N713	YD	2790189	3020612	230423	65
S505_N718	YD	5092785	5306771	213986	70
S505_N705	YD	5625352	5852470	227118	82
S511_N718	YD	5129056	5313969	184913	66
