# Supplementary material for: De novo assembly of a young Drosophila Y chromosome using single-molecule sequencing and chromatin conformation capture
Source: PLoS Biol. 2018 Jul 30;16(7):e2006348. doi: 10.1371/journal.pbio.2006348 (PMC6117089; doi:10.1371/journal.pbio.2006348)
Supplement: S7 Table — (PDF) [file pbio.2006348.s026.pdf]

S7 Table. Comparison of current and previous assembly of *D. miranda*

	Current *D. miranda* assembly (D.mir2.0)				D.mir1.0 (Zhou&Bachtrog 2012)*			
	Size in Mb	No. Scaffolds	Repeat %	Peri-centromere in Mb	Size in Mb	No. of Scaffolds	Repeat %	Peri-centromere in Mb
Total Genome	287.2	102	43.72	40.65	161.7	41,822	?	0.5
XL	25.3	1	18.27	0.55	22.1	1463	7.15	~0
XR	52.4	1	38.7	20	30.1	784	5.29	~0
Chr2	35.3	1	12.65	2	33	947	5.84	~0
Chr4	32.5	1	15.66	3.7	28.8	834	4.8	~0
dot	2.4	1	48.69	1.2	1.8	238	22.06	0.5
Neo-X	25.3	1	20.86	4	20.9	744	6.13	~0
Neo-Y	90.8	2	71.9	9.2	22	36,282	?	?
Unmapped	23.2	94	NA	NA	3	530	NA	NA

* Note that this comparison involves the stitched female genome only plus the neo-Y scaffolds
