# Supplementary material for: De novo assembly of a young Drosophila Y chromosome using single-molecule sequencing and chromatin conformation capture
Source: PLoS Biol. 2018 Jul 30;16(7):e2006348. doi: 10.1371/journal.pbio.2006348 (PMC6117089; doi:10.1371/journal.pbio.2006348)
Supplement: S8 Table — BUSCO, Benchmarking Universal Single-Copy Orthologs. (PDF) [file pbio.2006348.s027.pdf]

S8 Table. BUSCO analysis of assembly

Assembly stage →

BUSCO Scores	Canu assembly	Polished Falcon assembly	Double merged Canu and Falcon assembly	Hi-C-stitched Assembly	Final Assembly after corrections and polishing
Complete BUSCOS (C)	1057	1056	1058	1058	1060
Compete and Single copy BUSCOS (S)	897	900	902	911	908
Complete and Duplicated BUSCOS (D)	160	156	156	147	152
Fragmented BUSCOS (F)	1	1	1	1	1
Missing Buscos BUSCOS (M)	8	9	7	7	5

Assembly stage	BUSCO Scores
Canu assembly	C:99.1%[S:84.1%,D:15%],F:0.1%,M:0.8%,n:1066
Polished Falcon assembly	C:99%[S:84.4%,D:14.6%],F:0.1%,M:0.9%,n:1066
Double merged Canu and Falcon assembly	C:99.2%[S:84.6%,D:14.6%],F:0.1%,M:0.7%,n:1066
Hi-C-stitched Assembly	C:99.3%[S:85.5%,D:13.8%],F:0.1%,M:0.6%,n:1066
Final Assembly after corrections and polishing	C:99.5%[S:85.2%,D:14.3%],F:0.1%,M:0.4%,n:1066
Final Assembly after corrections and polishing with the 3 missing BUSCOS included	C:99.8%[S:85.5%,D:14.3%],F:0.1%,M:0.2%,n:1066

*3 BUSCOS reported as missing in the Final assembly were found to present in the MAKER annotation
