# Supplementary material for: De novo assembly of a young Drosophila Y chromosome using single-molecule sequencing and chromatin conformation capture
Source: PLoS Biol. 2018 Jul 30;16(7):e2006348. doi: 10.1371/journal.pbio.2006348 (PMC6117089; doi:10.1371/journal.pbio.2006348)
Supplement: S9 Table — A total of 90,977 molecules were obtained. (PDF) [file pbio.2006348.s028.pdf]

**S9 Table**. Molecule statistics for BioNano Data. A total of 90,977 molecules were obtained.

| | Min. | 1st Qu. | Median | Mean | 3rd Qu. | Max. | L50 | N50 |
|---|---|---|---|---|---|---|---|---|
| LabelChannel | 0 | 0 | 0 | 0 | 0 | 0 | 1 | 0 |
| MoleculeId | 127 | 539000 | 985500 | 919100 | 1296000 | 1817000 | 29141 | 1240544 |
| molLength | 150000 | 166800 | 191400 | 218700 | 235000 | 1957000 | 33991 | 209014.3 |
| molAvgIntensity | 0.02892 | 0.08189 | 0.1093 | 0.1158 | 0.1367 | 0.9999 | 31780 | 0.1 |
| labelSNR | 0.196 | 6.642 | 11.43 | 12.02 | 16.1 | 99.34 | 27308 | 15.1 |
| NumberofLabels | 1 | 11 | 16 | 17.99 | 22 | 249 | 26613 | 21 |
| OriginalMoleculeId | 2 | 15530 | 30130 | 33730 | 49250 | 92360 | 23582 | 48368 |
| ScanNumber | 1 | 5 | 11 | 10.69 | 17 | 20 | 26773 | 16 |
| ScanDirection | -1 | -1 | -1 | -1 | -1 | -1 | 1 | -1 |
| ChipId | 90977 | character | character | 90977 | character | character | NA | NA |
| Flowcell | 2 | 2 | 2 | 2 | 2 | 2 | 45489 | 2 |
| RunId | 1 | 1 | 1 | 1.339 | 2 | 2 | 30463 | 2 |
| GlobalScanNumber | 1 | 12 | 18 | 17.48 | 23 | 40 | 30198 | 21 |
| label100kdens | 0.1269 | 5.599 | 8.272 | 8.208 | 10.72 | 28.83 | 30433 | 9.8 |
