# Supplementary material for: De novo assembly of a young Drosophila Y chromosome using single-molecule sequencing and chromatin conformation capture
Source: PLoS Biol. 2018 Jul 30;16(7):e2006348. doi: 10.1371/journal.pbio.2006348 (PMC6117089; doi:10.1371/journal.pbio.2006348)
Supplement: S10 Table — (PDF) [file pbio.2006348.s029.pdf]

S10 Table. cDNA libraries used for annotation

Tissue/Sample	Number of read pairs	Total amount of data (no. of bases)
Male head	22547292	4.5Gb
Male whole body	211804052	42.8Gb
Male carcass	13682311	2.1Gb
Male testis	13522671	2.05Gb
Male accessory glands	28794285	4.4Gb
Male 3rd instar larvae	244994782	48Gb
Female head	20232892	4.04Gb
Female whole	25615616	5.17Gb
Female carcass	33564923	5.1Gb
Female ovary	32719471	4.97Gb
Female spermatheca	28924822	5.8Gb
Female 3rd instar larvae	64840245	12.97Gb
